# A human lectin array for characterizing host-pathogen interactions

**DOI:** 10.1016/j.jbc.2024.107869

**Published:** 2024-10-09

**Authors:** Stefi V. Benjamin, Sabine A.F. Jégouzo, Chloe Lieng, Connor Daniels, Marine Coispeau, Rikin J. Lau, Suyeon Kim, Yasmine Metaxa, James Philpott, Tiannuo Li, Chao Dai, Xin Wang, Maddy L. Newby, Gerald B. Pier, Max Crispin, Abigail Clements, Maureen E. Taylor, Kurt Drickamer

**Affiliations:** 1 Department of Life Sciences, Imperial College London, London, United Kingdom; 2 School of Biological Sciences, University of Southampton, United Kingdom; 3 Brigham and Women's Hospital, Harvard Medical School, Boston, Massachusetts, USA

**Keywords:** glycobiology, lectin, carbohydrate-binding protein, innate immunity, host-pathogen interaction, array screening, glycan-binding receptors

## Abstract

A human lectin array has been developed to probe the interactions of innate immune receptors with pathogenic and commensal microorganisms. Following the successful introduction of a lectin array containing all of the cow C-type carbohydrate-recognition domains (CRDs), a human array described here contains the C-type CRDs as well as CRDs from other classes of sugar-binding receptors, including galectins, siglecs, R-type CRDs, ficolins, intelectins, and chitinase-like lectins. The array is constructed with CRDs modified with single-site biotin tags, ensuring that the sugar-binding sites in CRDs are displayed on a streptavidin-coated surface in a defined orientation and are accessible to the surfaces of microbes. A common approach used for expression and display of CRDs from all of the different structural categories of glycan-binding receptors allows comparisons across lectin families. In addition to previously documented protocols for binding of fluorescently labeled bacteria, methods have been developed for detecting unlabeled bacteria bound to the array by counter-staining with DNA-binding dye. Screening has also been undertaken with viral glycoproteins and bacterial and fungal polysaccharides. The array provides an unbiased screen for sugar ligands that interact with receptors and many show binding not anticipated from earlier studies. For example, some of the galectins bind with high affinity to bacterial glycans that lack lactose or *N*-acetyllactosamine. The results demonstrate the utility of the human lectin array for providing a unique overview of the interactions of multiple classes of glycan-binding proteins in the innate immune system with different types of microorganisms.

One of the best-characterized functions of mammalian sugar-binding proteins, known as lectins, is recognition of bacteria and viruses. Many mammalian lectins are membrane receptors and soluble proteins that can target the innate immune response to pathogens, although this function is sometimes subverted by microorganisms to gain access to tissues and cells (1, 2). In this context, binding to sugars on microorganisms provides a means of distinguishing self from nonself. However, lectins can also target endogenous glycans, leading to turnover and clearance of glycoproteins and adhesion between cells.

Glycans bound by individual lectins have been extensively investigated. These studies have been facilitated by screening of glycan arrays with fluorescently labeled lectins to probe panels of immobilized glycans, which allows testing of hundreds of potential interactions in one experiment (3, 4). Most glycan arrays consist of mammalian glycans and are thus best suited for studying mammalian lectin interactions with host cells and endogenous glycoproteins. Arrays of bacterial glycans are available, but these arrays represent only a portion of the universe of glycans on microorganisms (5, 6, 7).

Lectin arrays, in which panels of immobilized lectins are probed with fluorescently labeled microorganisms, represent an alternative approach to identifying binding partners for the mammalian lectins. Arrays consisting largely of plant lectins were originally developed as a means of characterizing the structures of glycans such as those attached to glycoproteins (8, 9). More recently, an array containing immobilized cow C-type lectins has been used to screen for binding of fluorescently labeled bacteria, revealing overlapping patterns of interactions, some of which are not readily explained based on structures of known glycans on these organisms and previously characterized sugar-binding selectivity of the lectins (10). Such arrays provide an unbiased screen for sugar ligands that interact with receptors.

The cow array consisted entirely of members of one structural family of mammalian glycan-binding proteins, the C-type lectins. There are orthologs of many of the C-type lectins across mammalian species, so that results with the cow array can be used to suggest possible functions of related human proteins. However, there are also some lectins found in cows but not in humans, such as conglutinin and several other members of the serum collectin family, and other receptors such as L-SIGN (DC-SIGNR), prolectin and blood dendritic cell antigen 2 (BDCA-2) are found in humans but not in cows. In addition, while the C-type lectins are the most diverse of the lectin families, there are numerous other structural groups. The galectins and siglecs are the largest additional groups of lectin, but there are also other types of sugar-binding proteins with structurally distinct sugar-binding domains, including intelectins, ficolins, chitinase-type lectins, and proteins with R-type carbohydrate-recognition domains (CRDs) (11).

Based on the success of the cow array, construction of a human lectin array containing 39 different human CRDs from 36 sugar-binding receptors representing seven different structural groups has been constructed, and novel methods for detection of labeled and unlabeled microbes and polysaccharides are described. The results of screening with bacteria, viral glycoproteins, and polysaccharides from bacteria and fungi confirm known targets of the lectins but also reveal unexpected interactions of some of the lectins.

## Results

### A common approach to expression and display of CRDs

**Figure 1:**
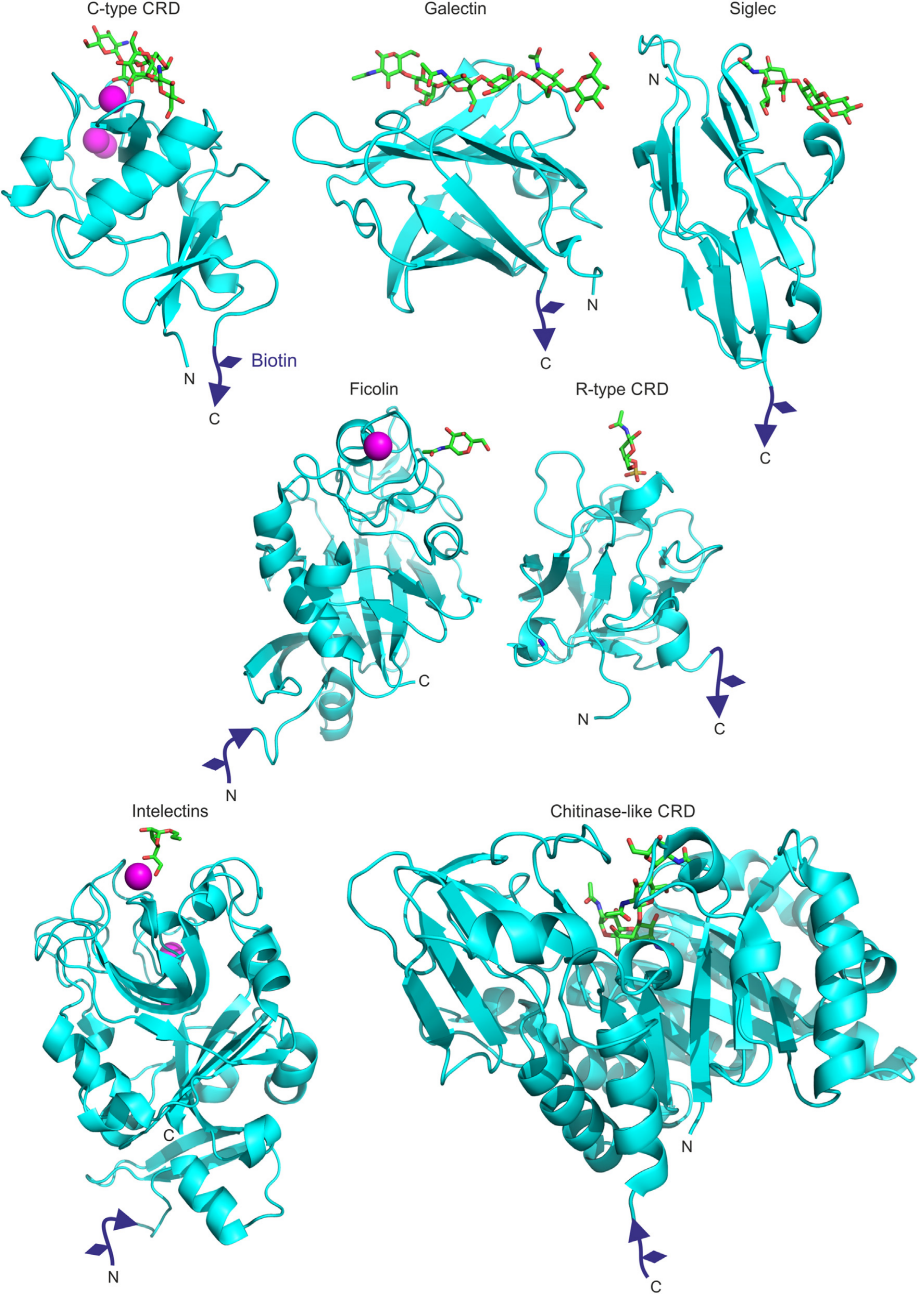
**Attachment of biotin tags to CRDs from different structural groups.** Representative structures for each group are shown: C-type CRD from DC-SIGN with Man_3_GlcNAc_2_ oligosaccharide (PDB 1K9I); galectin-9 with *N*-acetyllactosamine trimer (PDB 2ZHM); siglec 5 CRD with 3′-sialyllactose (PDB 2ZG3); ficolin 1 CRD with GalNAc (PDB 2JHI); R-type CRD from the mannose receptor with 4-sulfo-*N*-acetylgalactosamine (PDB 1DQO); intelectin 1 with allyl-β-galactofuranose (PDB 4WMY); and chitinase-like lectin Chi3L2 with (GlcNAcβ1-4)_4_ oligosaccharide (PDB 4P8W). Figure was generated with PyMol based on the indicated Protein Data Bank (PDB) files. Sugar ligands are shown as *sticks*, with carbon atoms in *green*, oxygen atoms in *red*, and nitrogen atoms in *blue*. Ca^2+^ is shown as *magenta spheres* and the appended biotin tags are in *purple*. CRD, carbohydrate-recognition domain; PDB, Protein Data Bank.

CRDs on the current version of the human lectin array are summarized in Table 1. Following the approach used in the cow array, human C-type CRDs were expressed with biotinylation tags using a bacterial system in which coexpressed biotin ligase catalyzes conjugation of biotin to a lysine residue in the tag appended at the C-terminal end of the CRD. Similar approaches were used for members of other structural families of human lectins (Fig. 1). Galectins that contain a single CRD were expressed as full-length proteins, except for galectin-3, which was truncated to remove an N-terminal extension. The two CRDs in tandem galectins were each expressed separately. Biotinylation tags were again appended at the C termini. Both the C-type CRDs and the galectin CRDs have loop-out topology, so the N and C termini are close to each other and are on the opposite side of the domain from the sugar-binding site. Test constructs, in which the tag was placed at the N terminus, showed identical binding properties.

**Table 1:** CRDs displayed on human lectin array

Abbreviation	Protein	Gene
MBP	Mannose-binding protein/lectin	MBL2
SP-A	Surfactant protein A	SFTPA1
SP-D	Surfactant protein D	SFTPD
ColK1	Collectin K1	COLEC11
MMR CRD 4	Mannose receptor/CD206 C-type CRD 4	MRC1
Langerin	Langerin	CD207
DC-SIGN	DC-SIGN/CD209	CD209
DC-SIGNR	DC-SIGNR/L-SIGN/CD299	CLEC4M
Prolectin	Prolectin	CLEC17A
LSECtin	LSECtin	CLEC4G
Endo180 CRD 2	Endo180/UPARAP C-type CRD 2	MRC2
Mincle	Mincle	CLEC4E
Dectin-2	Dectin-2	CLEC6A
BDCA-2	Blood dendritic cell antigen 2	CLEC4C
Dectin-1	Dectin-1	CLEC7A
ASGPR1	Asialoglycoprotein receptor subunit 1	ASGR1
ASGPR2	Asialoglycoprotein receptor subunit 2	ASGR2
MGL	Macrophage galactose receptor	CLEC10A
SRCL	Scavenger receptor C-type lectin	COLEC12
Galectin-1	Galectin 1	LGALS1
Galectin-2	Galectin 2	LGALS2
Galectin-3	Galectin 3	LGALS3
Galectin-7	Galectin 7	LGALS7
Galectin-4N	Galectin 4 N-terminal CRD	LGALS4
Galectin-4C	Galectin 4 C-terminal CRD	LGALS4
Galectin-8C	Galectin 8 C-terminal CRD	LGALS8
Galectin-9N	Galectin 9 N-terminal CRD	LGALS9
Galectin-9C	Galectin 9 C-terminal CRD	LGALS9
Siglec-1	Sialoadhesin	SIGLEC1
Siglec-3	CD33	CD33
Siglec-5	Siglec 5	SIGLEC5
Siglec-7	Siglec 7	SIGLEC7
Siglec-9	Siglec 9	SIGLEC9
Siglec-11	Siglec 11	SIGLEC11
Intelectin-1	Intelectin 1	ITLN1
Intelectin-2	Intelectin 2	ITLN2
MMR-R	Mannose receptor R-type CRD	MRC1
Ficolin 1	Ficolin 1/Ficolin M	FCN1
Chl3-L2	Chitinase 3-like lectin 2/YKL39	CHI3L2

Galectins differ from all of the other lectin families because the cysteine residues in the galectins are present as free sulfhydryl groups rather than forming disulfide bonds and in some cases, oxidation of the sulfhydryl groups leads to inactivation of galectins (12). Therefore, these cysteine residues were substituted with serine residues, except in galectin 2, where the single cysteine residue was changed to methionine in line with previous mutagenesis studies (13) and the C-terminal CRD of galectin 9 in which one cysteine was retained. The cysteine mutations in galectins 1, 2 and 3 have all been previously made and shown to have unchanged sugar-binding characteristics (12, 14, 15) and the natural form of galectin 4N contains no cysteine residues. One cysteine residue in galectin 9C has also been previously changed to serine without an effect on binding (16). Cysteine residues are found in analogous positions in galectins 4C and 8C. Similarly, one cysteine residue in galectin 9N is in an analogous position to one of the galectin 1 cysteine residues and an additional cysteine residue in galectin 9N has been previously changed to serine (16). Each of the remaining cysteine residues in galectins 7 and 9C are at least 10 Å away from the bound sugar in crystal structures of these CRDs (17, 18).

The CRDs in both the ficolins and the R-type CRD from the mannose receptor also have loop-out topology and biotinylation tags were again placed at the C-terminal ends of these domains. In contrast, the immunoglobulin-like domains in the siglecs have a pass-through topology, which places the N terminus near to the sugar-binding site. In each siglec, the sugar-binding V-set immunoglobulin-type domain is located at the N terminus, so the N terminus represents the beginning of the polypeptide, and the biotinylation tags were appended at the C termini. This arrangement again places the tag opposite to the sugar-binding site. However, in the full-length siglecs, one cysteine residue in the V-set domain is linked to a cysteine residue in an adjacent C-set immunoglobulin domain. For expression of the isolated CRD, this cysteine residue was changed to a serine residue (19). Although the intelectin and chitinase-like CRDs have loop-out topology, their structures suggested that the N termini would be more exposed than the C termini and are located on the opposite side from the sugar-binding site, so in each of these cases the biotinylation tag was placed at the N terminus (20, 21). In both cases, cysteine residues that would form interchain disulfide bonds in oligomers of the CRDs were changed to serine residues.

The CRDs were expressed in bacteria which allow efficient protein production and attachment of the biotin tag as well as eliminating potentially confounding effects of glycosylation on the CRDs themselves. Most of the CRDs were expressed as inclusion bodies, which were dissolved in guanidine and renatured. However, the galectins could be expressed directly in folded form in the cytoplasm of the bacteria. The chitinase-like lectin Chi3L2 was also expressed directly in folded form using bacteria that allow disulfide bond formation in the cytoplasm (22) and a bacterial signal sequence was used to direct the R-type CRD from the mannose receptor to the bacterial periplasm for folding. Correct folding of CRDs was demonstrated by purification on affinity columns constructed with appropriate sugar ligands and by binding of test ligands to the CRDs immobilized in streptavidin-coated wells. Test binding assays were also used to determine concentrations of protein needed to saturate the available biotin-binding sites in the wells. Although only some of the CRDs require Ca^2+^, test binding experiments confirmed that the presence of 2.5 mM Ca^2+^ did not affect binding to any of the other CRDs.

The criteria used for selection of lectins for inclusion on the array were that there is published evidence for involvement of the lectin or its close homologs in binding to pathogens and that sugar-binding activity can be demonstrated when possible by affinity chromatography on glycan ligand and always by binding of glycan ligands to immobilized CRDs. The array in its current form includes all of the human C-type lectins that are known to bind glycan ligands except for the three selectins, which were not included because they have not been reported to bind pathogen ligands. Their roles are in cell adhesion and not in immune recognition and clearance (23).

Among the galectins, galectin-12 and the N-terminal domain of galectin-8 have not been included in the array. The C-terminal domain of galectin-12 lacks most of the conserved binding sites residues found in the other galectins (24, 25). Although weak affinity for lactose has been reported for the N-terminal domain, bacterially expressed, biotin-tagged protein did not bind to a lactose affinity column. Galectin-8 N-terminal domain was successfully purified on lactose-Sepharose, but binding of glycan ligands to the immobilized CRD was difficult to establish. In the absence of demonstrated activity in the array format as positive controls, data for these proteins have not been included.

Although the human siglecs primarily mediate signaling initiated by endogenous human glycans, representative siglecs have been included in the array because some viruses and other microorganisms co-opt these receptors (26, 27). The CRDs expressed are from siglecs that bind α2-3, α2-6, and α2-8 linked sialic acid. Siglecs not on the array are siglec-8, which has unique specificity for sulfated ligands that has proven difficult to confirm in the expressed CRD, siglec-4, which is not expressed in the immune system, siglecs-14 and 16, which have CRDs identical to siglecs-5 and 11, and siglecs-2, 6, 10, and 15 which have binding specificities that overlap with CRDs that are on the array (28).

All three human ficolins have GlcNAc-related sugar-binding activity, although defined oligosaccharide ligands have not been extensively characterized (29). Because the sugar-binding activity of ficolin-1 could be confirmed by affinity chromatography on immobilized GlcNAc, it was chosen as a representative example. Similarly, all three chitinase-like lectins bind to chitin and Chi3L1 and Chi3L2 binding to immobilized chitin oligosaccharides has been confirmed in glycan-array analysis (30, 31). Because Chi3L2 could be purified by affinity chromatography on chitin oligosaccharides, it was used as the representative example.

The final version of the array used for screening contains CRDs from the pathogen-binding C-type lectins, both of the intelectins, the R-type CRD of the mannose receptor, all of the galectins except galectin 8N and 12, as well as the representative members of the siglec, ficolin, and chitinase-like lectin families (Table 1).

### Probing with labeled bacteria

**Figure 2:**
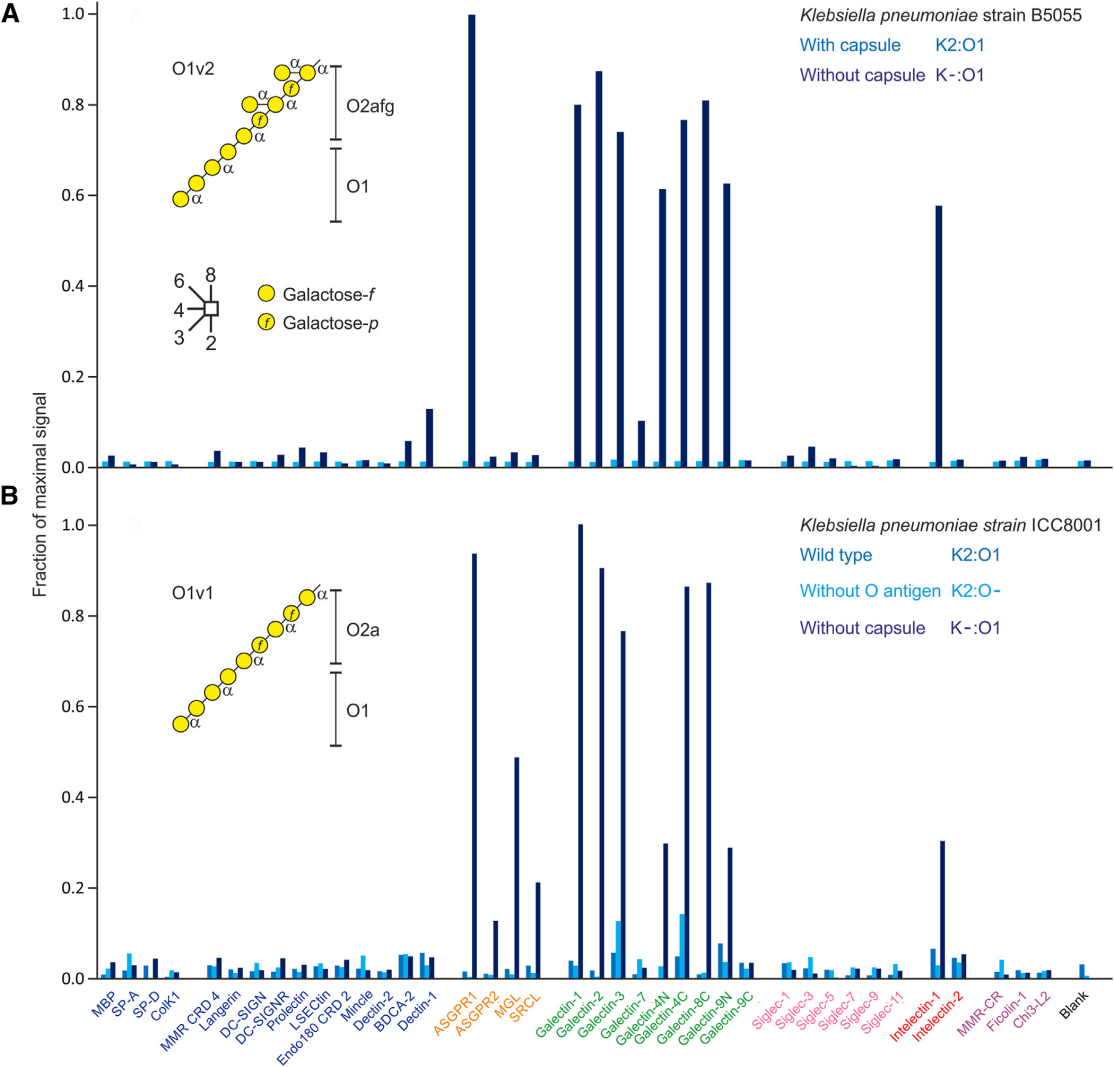
**Interaction of *Klebsiella pneumoniae* with lectins on the array.** Human lectin array was screened with versions of two strains of *K. pneumoniae* with and without capsules. Cells expressing GFP were grown to stationary phase and fixed with paraformaldehyde. Results were normalized to the maximum signal for the unencapsulated strain in each panel. For each strain, a representative example from three experiments is shown. *A*, screening with strain B5055, serotype K2:O1v1, and a mutant lacking the capsule at concentrations of 2.5 to 5 × 10^8^ cells/ml. The structure of the O1v1 outer polysaccharide is shown. Average errors were 12%. *B*, screening with strain ICC8001, serotype K2:O1v2, and mutants lacking either the capsule or the O-antigen at concentrations of 5 to 10 × 10^8^ cells/ml. The structure of the O1v2 outer polysaccharide is shown. Average errors were 10%. Data are reported in Table S1.

Screening of the cow lectin array was undertaken with fluorescently labeled bacteria, which were either expressing GFP or were chemically labeled with fluorescein fluorophores (10). A similar approach with the human array, comparing binding of *Klebsiella pneumoniae* strains with and without capsular polysaccharides, shows that binding to the array increases dramatically in the absence of the capsule (Fig. 2). The results demonstrate that the capsule effectively shields bacteria from all of the receptors tested. It is likely that binding seen in the absence of the capsule is to exposed O1 antigen, which in the strains tested consists of both α- and β-linked galactopyranose and galactofuranose residues (32, 33, 34). Terminal galactopyranose residues can interact with the asialoglycoprotein receptor (10) and galactofuranose residues present in the O-oligosaccharide are a preferred target for intelectin 1 (20). Several galectins also bind when the O-antigen is exposed. Binding of galectins 3, 4, 7, 8 and 9 to *Klebsiella* with O1 oligosaccharides has previously been attributed to the presence of Galα1-3Gal units (5, 35, 36). The array results are largely consistent with these findings, although galectin 7 binding is low. Two different strains of K2:O1 *Klebsiella*, shown in Figure 2, *A* and *B*, display the same overall pattern, but there are some differences that may reflect different proportions of the different forms of galactose or differences in accessibility of the terminal structures in the different versions of the O antigen.

**Figure 3:**
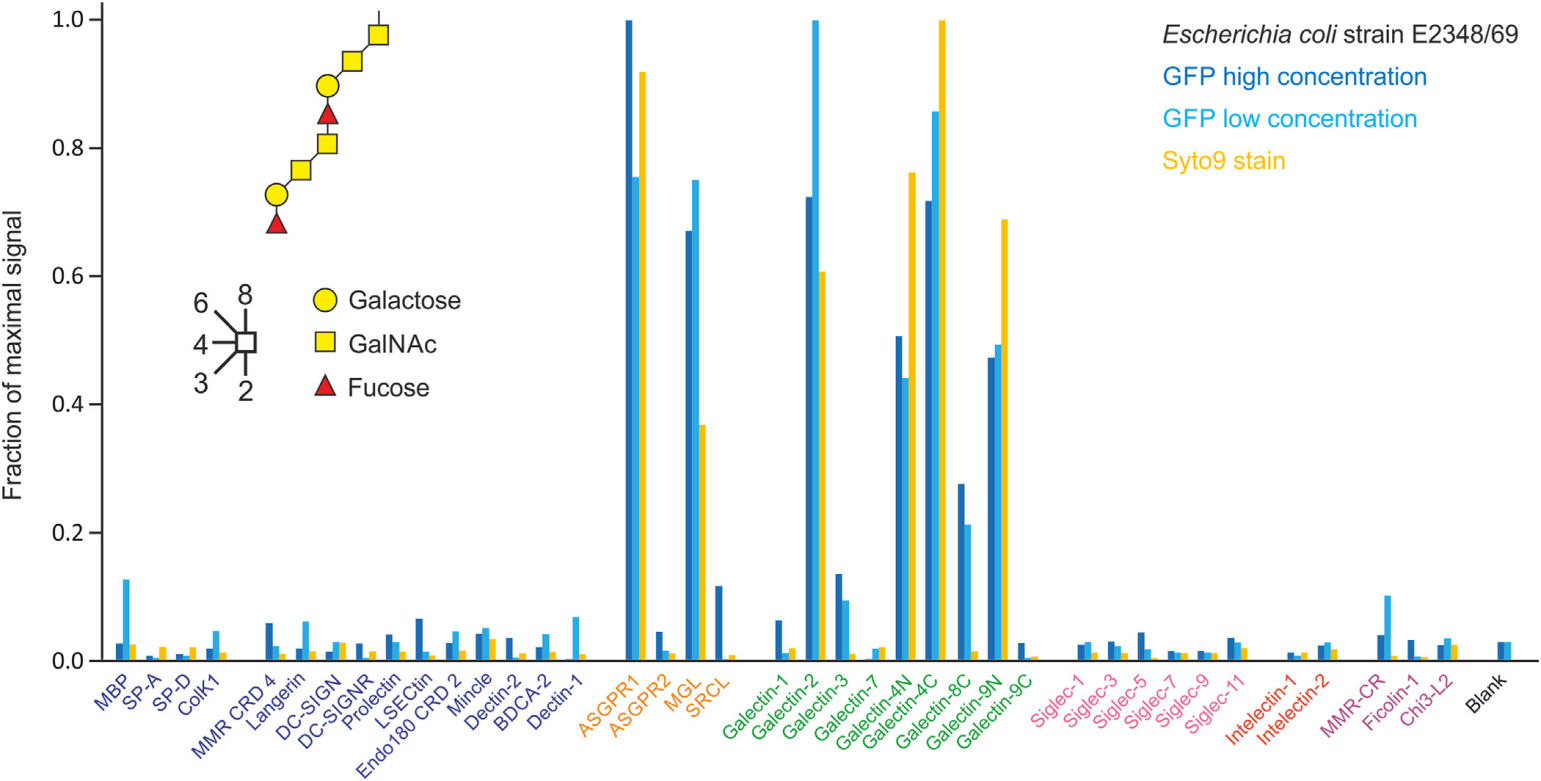
**Enteropathogenic *Escherichia coli* binding to the human lectin array.** Enteropathogenic *E. coli* strain E2348/69 (O127:H6) grown to stationary phase and fixed with paraformaldehyde were used to screen the array. Cells expressing GFP were screened at 1.6 × 10^8^ cells/ml (low concentration) and 8 × 10^8^ cells/ml (high concentration). Cells without GFP were screened at 16 × 10^8^ cells/ml and were visualized by counter-staining with Syto 9 dye. Average errors were 10%, 9%, and 3% for the three different protocols. A representative example from two experiments is shown. Results for each experiment were normalized to the highest signal. Structure of two repeat units from the O127 outer polysaccharide of lipopolysaccharide is shown. Data are reported in Table S2.

Screening of the human array with an enteropathogenic strain of *Escherichia coli* was used to demonstrate detection of bound bacteria without the need for prior labeling. Unlabeled bacteria were bound in the usual way, followed by staining with the membrane-permeable DNA-binding dye Syto 9. Syto 9 staining results in a higher background signal in uncomplexed wells, but the background signal is constant and can be subtracted to yield results that are comparable to binding of GFP-labeled bacteria (Fig. 3). The O127 outer polysaccharide contains galactose residues with exposed 3- and 4-OH groups, which is consistent with the observed binding to the major subunit of the asialoglycoprotein receptor and the macrophage galactose receptor (37, 38). The additional binding to several galectins represents a departure from the usual lactose-type ligands for the galectins. However, the disaccharide Galβ1-3GalNAc found in the repeat unit has been shown to be a ligand for galectins 4 and 9 (39, 40) and the Fucα1-2Galβ1-3GalNAc (H-antigen) structure has also been shown to bind galectin 4 (41).

### Probing with bacterial polysaccharides

**Figure 4:**
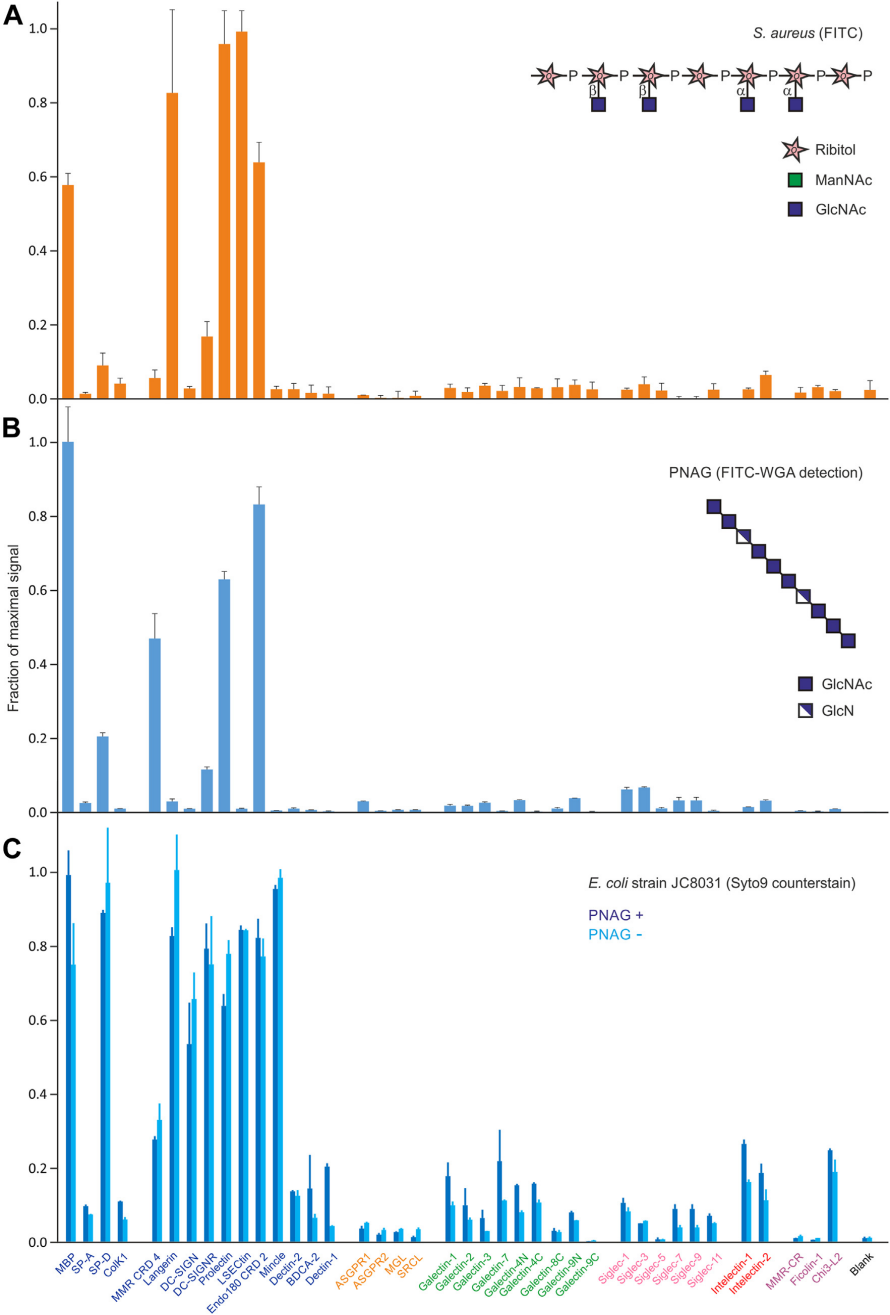
**Screening of human lectin array with *Staphylococcus aureus* and PNAG.***A*, screening with FITC-labeled, heat-killed cells of the Wood 46 strain of *S. aureus* at a concentration of 1 × 10^8^ cells/ml. Average errors were 13%. *B*, screening with PNAG at 60 μg/ml, followed by visualization with FITC-labeled wheat germ agglutinin at 40 μg/ml. Average errors were 7%. *C*, screening with *Escherichia coli* strain *E. coli* JC8031 and a mutant lacking PNAG at 4 to 12 × 10^8^ cells/ml. Bound cells were counterstained with Syto 9 dye. Average errors were 10%. Results were normalized to the highest signal in each experiment. Structures of wall teichoic acid from *S. aureus*, showing α-linked GlcNAc residues, and of PNAG are shown. A representative example from two experiments is shown. Data are reported in Table S3. PNAG, poly-*N-*acetylglucosamine.

Fluorescein-labeled *Staphylococcus aureus* binds only to a restricted subset of C-type CRDs and not to CRDs in other families (Fig. 4*A*). Wall teichoic acid on the surface of the Wood strain bears α- and β-linked GlcNAc residues, which are likely to target the binding of these CRDs (42). The CRD from Endo180 specifically targets GlcNAc residues and the CRD of mannose-binding protein is not sensitive to the position of 2-substituents on sugars, so it binds GlcNAc as well as mannose residues (43, 44). Human langerin, prolectin, and LSECtin also bind GlcNAc and GlcNAc-containing oligosaccharides (45, 46, 47). Binding to LSECtin is mostly associated with GlcNAc linked to mannose residues, but the array results suggest that it can bind GlcNAc in other linkages, in this case to ribitol phosphate.

In addition to the O-antigen polysaccharides on lipopolysaccharides and capsular polysaccharides, potential targets for lectin binding on bacteria include exopolysaccharides that can be cell bound or secreted into the extracellular space to provide a matrix on which biofilms are organized (48). Although the matrix may help to shield bacteria from the immune system, components of the matrix can themselves be targets for the immune system. To demonstrate the feasibility of screening for interactions of a polysaccharide surface and biofilm components with the innate immune system, the human lectin array was probed with purified poly-*N-*acetylglucosamine (PNAG, (GlcNAcβ1-6GlcNAc)_n_) isolated from *Acinetobacter baumannii* (49).

Lectin-bound PNAG could be detected by secondary binding of fluorescently labeled wheat germ agglutinin (Fig. 4*B*), although comparable results were observed with PNAG in which free amino groups were labeled with fluorescein (data not shown). Binding was observed exclusively to C-type lectins that bind GlcNAc, which partially overlaps the binding to lipoteichoic acid on *S. aureus* and peptidoglycan expressed by prokaryotes. The major difference in presentation of GlcNAc residues in staphylococcal teichoic acids with peptidoglycan and PNAG is that, rather than being exposed at nonreducing termini, the sugars in peptidoglycan and PNAG are mostly internal. The 1 to 6 linkage in PNAG leaves the 3- and 4-OH groups of GlcNAc exposed, and it is these groups that form the primary interactions with primary binding sites in C-type CRDs (38). Nevertheless, the 1 to 6 linkage appears to interfere with binding to LSECtin and langerin. In contrast, binding to the CRD4 of the macrophage mannose receptor is enhanced, which suggests that in this case there may be secondary interactions of adjacent sugar residues in the PNAG chain.

Additional experiments were undertaken with isogenic strains of bacteria with and without a functional pga gene needed for synthesis of PNAG (Fig. 4*C*). Although these cells bound to all of the receptors that bind PNAG, they also bind to additional receptors and there is no clear difference between binding of strains with and without functional *pga* genes. Similar results were obtained with cells fixed with methanol to stabilize PNAG. These results indicate that binding to other surface polysaccharides such as peptidoglycan can occur in the absence of PNAG expression, suggesting that interaction of the lectins with a PNAG-based biofilm matrix may be more robust than binding to the bacteria *via* PNAG.

### Probing with fungal polysaccharide

**Figure 5:**
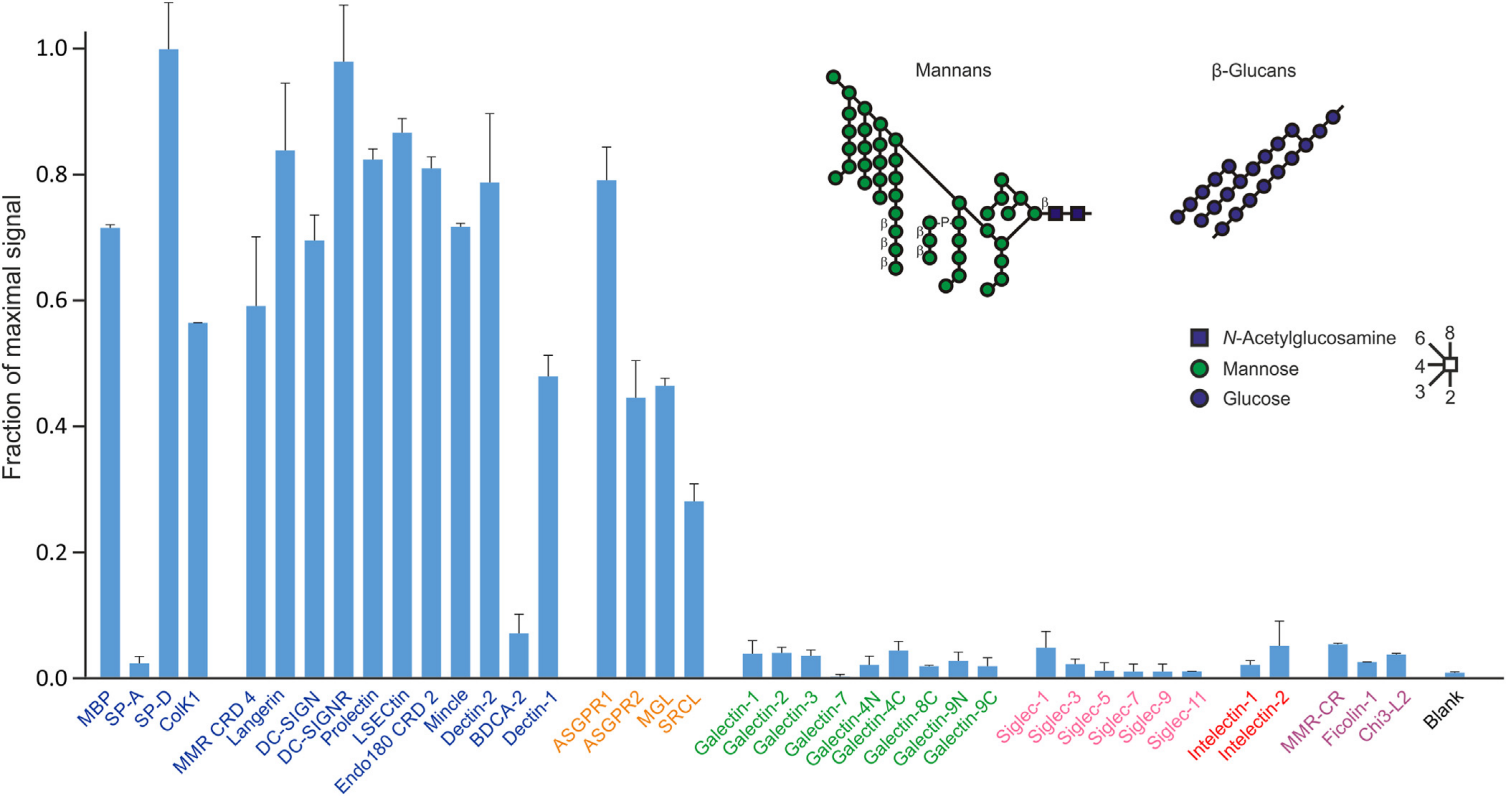
**Binding of yeast zymosan to human lectin array.** FITC-labeled zymosan was used for screening at a concentration of 1 × 10^7^ particles/ml. Average errors were 7%. A representative example from two experiments is shown. Structures of the predominant yeast wall mannans and β-glucans in zymosan are shown. Ligands for galactose-binding receptors are also present, but the relevant structures are not known (66). Data are reported in Table S4.

Screening with fluorescein-labeled yeast zymosan demonstrated binding to most of the C-type CRDs on the array (Fig. 5). This pattern is consistent with the results observed for the cow array, reflecting the presence of polysaccharides containing mannose, glucose, and galactose in the yeast cell wall. The C-type CRDs which do not show binding are pulmonary surfactant protein SP-A, which has weak affinity, and BDCA-2, which has very restricted specificity for ligands containing galactose linked to GlcNAcβ1-2Man disaccharide (50, 51). Similarly, the absence of binding to any of the other lectin families is consistent with the absence of target ligands, such as lactose and sialic acid in this preparation.

### Probing with viral glycoproteins

Unlike the glycans expressed on bacteria and fungi, the glycans on viruses are generated by the host glycosylation machinery. Although the resulting overlap between glycans on viruses and host cells can make it difficult to distinguish viruses as nonself by recognition of their surface glycans, selective expression of some types of glycans on viruses can be a strategy for targeting cells for infection (47, 52). The utility of the human lectin array for screening viral interactions was explored using surface glycoproteins from HIV and influenza virus. Glycoproteins with well-characterized glycan compositions were prepared in transfected cells and directly labeled with fluorophores (53, 54).

**Figure 6:**
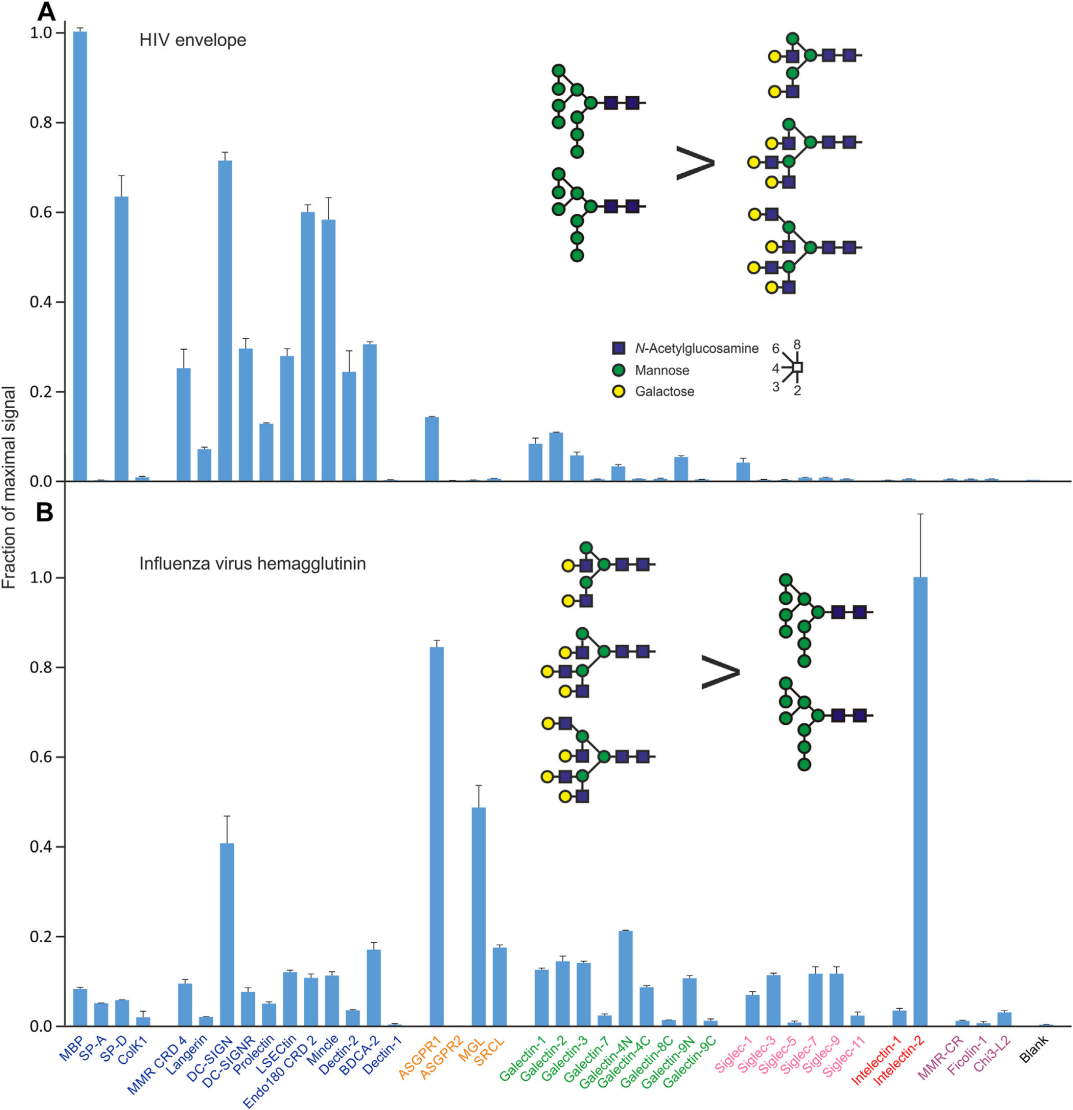
**Viral glycoproteins tested on human lectin array.** Soluble, trimeric forms of the surface glycoproteins were generated using trimerization sequences to replace the membrane anchors. Purified proteins were labeled with Alexa488 and used to screen the array. *A*, HIV BG505 SOSIP.664 Envelope glycoprotein (7.9 μg/ml). Average errors were 6%. *B*, influenza virus H3 Brisbane/2007 hemagglutinin (6.2 μg/ml). Average errors were 7%. For each protein, a representative example from three experiments, covering a 10-fold concentration range, is shown. Examples of potential target glycans are shown. Glycans present on HIV envelope protein are predominantly high mannose N-linked oligosaccharides with up to nine mannose residues. More glycans on influenza virus hemagglutinin are complex glycans, with variable numbers of branches, often terminating in galactose. Data are reported in Table S5.

The patterns of binding for the two viral proteins were strikingly different, reflecting their distinct glycan profiles (Fig. 6). The abundance of high mannose oligosaccharides on the HIV spike glycoprotein results in interaction with almost all of the C-type lectins that target mannose and related sugars. Although interaction with DC-SIGN has been particularly investigated as a mechanism for binding HIV to dendritic cells, these results confirm that the virus can interact with other receptors, which could also mediate binding to other types of cells.

The hemagglutinin of influenza virus interacts with a much broader range of receptors, including receptors that bind terminal galactose, GalNAc, and sialic acid as well as the mannose-binding receptors. This broad range of interactions reflects the more heterogeneous glycosylation of the influenza virus surface, including complex as well as high mannose N-linked glycans (54, 55). Among the galactose-type receptors, there is considerable variability in the extent of binding, including very high binding to intelectin 2, enhanced binding to galectin 3 compared to other galectins, and preferential binding to the macrophage galactose receptor compared to the closely related asialoglycoprotein receptor. Some binding to the scavenger receptor C-type lectin is also observed. This receptor is largely selective for the Lewis-x trisaccharide (56). Although it is not possible to directly account for all the details of the binding based on known ligands for the receptors and the glycan composition on the virus, these results provide an empirical demonstration of the range of potential interactions.

## Discussion

A human lectin array has been developed to probe the interactions of innate immune receptors with microorganisms. Compared to an earlier array of cow C-type CRDs, the human array described here contains CRDs from multiple structural groups: C-type CRDs, galectins, siglecs, ficolins, intelectins, an R-type CRD, and a chitinase-like lectin. The use of single-site biotin tags appended at the N or C termini of the CRDs ensures that the sugar-binding sites in CRDs are displayed on a streptavidin-coated surface in a defined orientation and are accessible to the surfaces of microbes. In addition to previously documented protocols for binding of fluorescently labeled microorganisms, protocols have been established for detecting unlabeled bacteria and fungi bound to the array by counter-staining with DNA stains. Screening has also been undertaken with fluorescently labeled viral glycoproteins and binding of bacterial polysaccharides can be detected by secondary binding of a fluorescently labeled plant lectin. The results demonstrate that a common approach can be used for expression and display of CRDs from all of the different structural categories of glycan-binding receptors, allowing comparisons across lectin families.

The lectin array provides an unbiased screen for sugar ligands that interact with receptors and many show binding not anticipated from earlier studies. For example, some receptors usually associated with binding of endogenous mammalian glycans, such as the asialoglycoprotein receptor, also bind to bacteria and some of the galectins bind to bacterial glycans that lack lactose or *N*-acetyllactosamine. Similarly, LSECtin binds well to yeast and bacteria that do not contain the GlcNAcβ1-2Man epitope identified as the common binding motif in glycan array screening of this receptor. Receptors in some groups, such as the galectins or the mannose-binding C-type lectins DC-SIGN, langerin, and the mannose receptor, bind many of the same microorganisms, but in each case there are some microorganisms that bind only to a subset of each group.

Potential targets for lectin binding include bacterial lipopolysaccharides, pilus glycoproteins, wall polysaccharides, and capsular polysaccharide. However, some of these targets can be masked in intact microorganisms, as demonstrated by exposure of galactose-containing ligands for C-type lectins, galectins, and intelectins in strains of *Klebsiella* that lack the capsular polysaccharide. Comparison of the results for strains with and without the capsule provide a clear demonstration of the effectiveness of the capsule in shielding bacteria from all of the sugar-binding receptors of the innate immune system that are present on the array.

The initial screening results presented here demonstrate the utility of the human lectin array in providing a unique overview of the interactions of multiple classes of glycan-binding proteins in the innate immune system with different types of microorganisms. When binding is not readily explained based on known structures on microorganisms, the biotin-tagged receptors can potentially be used as tools for identification and characterization of target ligands.

## Experimental procedures

### Expression systems

Expression and purification of biotin-tagged CRDs for the following proteins have been previously described: the scavenger receptor C-type lectin (56), mincle (57), dectin-2 (58), BDCA-2 (51), prolectin (46), and MMR-CRD4 (59). For the remaining proteins, regions encoding CRDs were selected based on previous expression studies and available crystal structures and complementary DNAs based on corresponding genomic sequences, modified to include biotinylation tags (60), were synthesized by GeneArt. The sequences, shown in Figs. S1–S33, were transferred to the expression vector.

The R-type CRD from the mannose receptor was expressed in plasmid pINIIIompA2 in *E. coli* strain JA221 as previously described for the untagged protein (43). All other CRDs were expressed from the T7 promotor in plasmid pT5T (61), in *E. coli* strain BL21(DE3) except for Chi3L2, which was expressed in SHuffle cells (New England Biolabs). These strains also carried plasmid pBirA, which encodes biotin ligase (62). Bacteria were grown with shaking in Luria-Bertani medium containing 50 μg/ml ampicillin and 25 μg/ml chloramphenicol at 37 °C. Two general expression protocols were used, as indicated in Table 2. All CRDs except the galectins, the R-type CRD from the mannose receptor and Chi3L2 were expressed at 37 °C, recovered from inclusion bodies by solubilization in guanidine, and renatured by one of the published protocols: fast renaturation by dilution into buffer or slow renaturation by dialysis against buffer (10). In some cases, renatured proteins were further dialyzed against water, lyophilized and taken up in a smaller volume of buffer before affinity chromatography. Mincle was renatured in the presence of 1% Triton X-100 and treated with Amberlite XAD-2 beads before dialysis against water and lyophilization.

**Table 2:** Protocols for expression of biotin-tagged CRDs

Protein	Extraction	Renaturation	Resin	Test ligands
MBP	Renaturation	Dilution	Mannose	Zymosan
SP-A (neck)	Renaturation	Dialysis/lyophilization	ManNAc	Zymosan
SP-D	Renaturation	Dialysis	Maltose	Zymosan
ColK1	Renaturation	Dialysis	Mannose	Zymosan
MMR CRD 4	Renaturation	Dialysis	Mannose	Zymosan
Langerin	Renaturation	Dialysis/lyophilization	Mannose	Zymosan
DC-SIGN	Renaturation	Dilution/lyophilization	Mannose	Zymosan
DC-SIGNR	Renaturation	Dilution/lyophilization	Mannose	Zymosan
Prolectin	Renaturation	Dilution	Mannose	Zymosan
LSECtin	Renaturation	Dialysis	Mannose	Zymosan
Endo180 CRD2	Renaturation	Dialysis	GlcNAc	PNAG
Mincle	Renaturation	Triton/lyophilization	Trehalose	Zymosan
Dectin-2	Renaturation	Dialysis	Mannose	Zymosan
BDCA-2	Renaturation	Dilution	Asialo egg GP	Asialofetuin
Dectin-1	Renaturation	Dialysis	MonoQ/S75	Zymosan
ASGPR1	Renaturation	Dilution	Galactose	Zymosan
ASGPR2	Renaturation	Dialysis/lyophilization	Galactose	Zymosan
MGL	Renaturation	Dilution	Galactose	Zymosan
SRCL	Renaturation	Dialysis	Galactose	Influenza HA
Galectin-1	Sonication	-	Lactose	*Klebsiella* cap^-^
Galectin-2	Sonication	-	Lactose	*Klebsiella* cap^-^
Galectin-3	Sonication	-	Lactose	*Klebsiella* cap^-^
Galectin-7	Sonication	-	Lactose	Asialofetuin
Galectin-4N	Sonication	-	Lactose	*Klebsiella* cap
Galectin-4C	Sonication	-	Lactose	*Klebsiella* cap^-^
Galectin-8C	Sonication	-	Lactose	*Klebsiella* cap^-^
Galectin-9N	Sonication	-	Lactose	*Klebsiella* cap^-^
Galectin-9C	Sonication	-	Lactose	Asialofetuin
Siglec-1	Renaturation	Dialysis	Fetuin	Ganglioside liposomes
Siglec-3	Renaturation	Dialysis	Fetuin	Ganglioside liposomes
Siglec-5	Renaturation	Dialysis	Fetuin	Ganglioside liposomes
Siglec-7	Renaturation	Dialysis	Fetuin	Ganglioside liposomes
Siglec-9	Renaturation	Dialysis	Fetuin	Ganglioside liposomes
Siglec-11	Renaturation	Dialysis/lyophilization	Fetuin	Ganglioside liposomes
Intelectin-1	Renaturation	Dilution/lyophilization	Galactose	Gal-BSA
Intelectin-2	Renaturation	Dialysis	Galactose	Gal-BSA
MMR-R	Sonication	-	Lutropin	Lutropin
Ficolin 1	Renaturation	Dialysis	GlcNAc	PNAG
Chl3-L2	Sonication	-	Chitin	*Candida albicans*

Methods for protein expression, extraction from bacteria, and affinity purification are indicated for each protein.

For direct expression of folded galectin and Chi3L2 CRDs, cells were grown at 37 °C to *A*_550_ of 0.4, at which point the temperature was reduced to 25 °C. At *A*_550_ of 0.7, isopropyl-β-D-thiogalactoside was added to a concentration of 100 μg/L and biotin was added to a concentration of 12.5 μg/L. The incubation was continued overnight. Bacteria were harvested by centrifuging at 4000*g* for 15 min at 4 °C, washed with Tris-buffered saline (TBS) and pelleted at 10,000*g* for 10 min at 4 °C. Cell pellets from 2 L of culture were sonicated in 50 ml of TBS 6 times for 30 s at full power using a Branson 250 sonicator, and centrifuged for 30 min at 20,000*g* in Beckman JA-14 rotor. Supernatants were applied directly to affinity columns.

Affinity columns used for purification were prepared either by divinyl sulfone coupling of monosaccharides, disaccharides, or oligosaccharides to Sepharose (63) or by coupling of glycopeptides or glycoproteins to Affigel-10 and 15 resins (Bio-Rad Laboratories). Preparations of immobilized bovine lutropin and desialylated egg yolk glycopeptide have been previously described (43, 47). Fetuin columns were made by coupling 500 mg of bovine fetuin (Sigma-Aldrich) in 15 ml of 100 mM Mops, pH 7.5, to 25 ml of Affigel-15 for 4 h at 4 °C and 30 min at room temperature.

For the C-type CRDs, columns were washed with 10 ml of 150 mM NaCl, 25 mM Tris–Cl, pH 7.8, 25 mM CaCl_2_ and eluted in 1-ml aliquots of 150 mM NaCl, 25 mM Tris–Cl, pH 7.8, 2.5 mM EDTA. The SP-A protein shows very weak binding, so an extended CRD containing part of the trimerization sequence was included in the construct. At high concentration, the resulting weak association was sufficient to allow purification of active protein on immobilized ManNAc, but the protein eluted as a monomer on gel filtration at dilute concentrations used for coating the plate. The CRD from dectin-1 could not be purified by affinity chromatography. Renatured protein was dialyzed against 50 mM Tris–Cl, pH 7.8, and loaded onto a 1-ml MonoQ ion exchange column, which was eluted with a gradient from 0 to 0.5 M NaCl containing 50 mM Tris–Cl, pH 7.8. Fractions containing the renatured CRDs, identified by SDS-PAGE, were pooled, dialyzed against water, and lyophilized before final purification on a Superdex S75 column run in 100 mM NaCl, 10 mM Tris–Cl, pH 7.8, 2.5 mM EDTA.

Intelectins were purified as for the C-type lectins, except that intelectin-2 was eluted with buffer containing 25 mM EDTA. Ficolin M was purified on GlcNAc-Sepharose and eluted with 0.5 M GlcNAc. Chi3L2 was purified on a mixture of β1-4 linked GlcNAc oligomers obtained by hydrolysis of chitin (64) and immobilized by the divinyl sulfone method. Chi3L2 was eluted with 2 M urea and dialyzed against TBS. The R-type CRD from the mannose receptor was purified as previously described (43). To ensure complete biotinylation, 1.5 mg of the purified protein was incubated with 15 μg of biotin ligase (Avidity) for 24 h at 30 °C following the manufacturer's protocol. The biotinylated protein was repurified on immobilized lutropin. Galectin CRDs were purified on lactose-Sepharose in TBS. After application of sample, columns were washed with 10 ml of TBS and eluted with 0.2 M lactose in TBS. Siglec CRDs were dialyzed against 25 mM Tris, pH 7.8, before binding to fetuin columns. Bound CRDs were eluted with 100 mM glycine, pH 2.2, which was immediately neutralized with 1 M Tris–Cl, pH 8.5. In all cases, fractions were examined on SDS-polyacrylamide gels containing 17.5% polyacrylamide.

### Labeled microorganisms

Bacterial strains used are summarized in Table 3. Fluorescein-labeled preparations of zymosan from *Saccharomyces cerevisiae*, *E. coli* K-12, and the Wood 46 strain of *S. aureus* were obtained from Molecular Probes (Thermo Fisher Scientific). Heat-killed *Candida albicans* cells were obtained from Invivogen. Cells were grown to stationary phase, washed in phosphate-buffered saline, fixed for 30 min in 5% paraformaldehyde in PBS, and washed with Tris-buffered saline. Bacteria were quantified by counting of suitable dilutions in a Helber counting chamber.

**Table 3:** Bacterial strains used in this study

Bacterial strain	Serotype	Plasmid
Enteropathogenic *Escherichia coli* strain E2348/69	O127:H6	pACYC184-GFP (67)
*Escherichia coli strain* JC8031		-
*Escherichia coli strain* JC8031 Δpga		-
*Klebsiella pneumoniae* strain ICC8001 (68)	K2:O1v1	pUltra-GFP/Gm (69)
*Klebsiella pneumoniae* strain ICC8001 ΔwaaL (70)	K2:O-	pUltra-GFP/Gm (69)
*Klebsiella pneumoniae* strain ICC8001 ΔwcaJ (70)	K-:O1	GFP inserted at chromosomal T7 site (68)
*Klebsiella pneumoniae* strain B5055	K2:O1v2	pUltra-GFP/Gm (69)
*Klebsiella pneumoniae* strain B5055 nm (71)	K-:O1	pUltra-GFP/Gm (69)
*Staphylococcus aureus* strain Wood		-

### Viral glycoproteins

Plasmids pPPI4 and pM for expression of HIV-1 envelope glycoprotein BG505 SOSIP.664 and influenza virus hemagglutinin H3 Brisbane/2007 were transiently transfected into human embryonic kidney 293F cells (53, 54). Cells were cultured in FreeStyle 293 Expression Medium (Thermo Fisher Scientific) and maintained at a density of 0.2 to 3 × 10^6^ cells/ml at 37 °C, 8% CO_2_, and 125 rpm shaking. Expression plasmid at 310 μg/L 25 ml in Opti-MEM (Thermo Fisher Scientific) was combined with a further 25 ml of Opti-MEM containing 930 mg/ml of pH 7 polyethylenimine max reagent and incubated for 30 min at room temperature. Cells were transfected at a density of 1 × 10^6^ cells/ml and incubated for 7 days. Cells were removed by centrifugation at 3000*g* for 30 min at 4 °C, and the supernatant was filtered through a 0.22 μm filter using a 500-mL Stericup-HV sterile vacuum filtration system (Merck). A 5-mL HisTrap Excel column charged with Ni(II) on an ÄKTA Pure system (Cytiva) was equilibrated using 10 column volumes of washing buffer (50 mM sodium phosphate, pH 7.0, 300 mM NaCl). The filtered supernatant was loaded at a flow rate of 5 ml/min and the column was washed with 10 column volumes of 50 mM imidazole in washing buffer. Protein was eluted in three column volumes of 300 mM imidazole in washing buffer, exchanged into phosphate-buffered saline, concentrated to 1 ml using a 100 kDa cut-off Vivaspin, and run on a Superdex 200 pg 16/600 column (Cytiva) eluted with PBS at 1 ml/min. Purified trimers were concentrated to 1 mg/ml and were conjugated to Alexa Fluor 488 dye using a protein labeling kt (Invitrogen) following the manufacturer’s instructions.

### PNAG

PNAG was purified from *A. baumannii* overexpressing the bacterial *pga* genes encoding the biosynthetic enzymes as described (49). Briefly, arabinose-induced cells were grown in Luria-Bertani broth for 72 h at 37 °C, the recovered bacterial cells sequentially treated with lysozyme, followed by DNase and RNase, after which cell bodies were sedimented by centrifugation. PNAG in the supernatant was precipitated by addition of three volumes of 95% ethanol. The insoluble PNAG was recovered, suspended in water, dialyzed against water overnight at 4 °C, and lyophilized. The purified material was quantified (65) and characterized by ^1^H- and ^1^H-^1^H correlation spectroscopy (COSY) NMR (49). PNAG was solubilized by first dissolving in 20 to 100 μl of 5 M HCl followed by addition of an equal volume of 5 M NaOH before dilution in neutral pH buffer to the working concentration used in the assays.

### Glycoproteins and liposomes

For fluorescein labeling, proteins (1 mg) were reacted with 12.5 μg fluorescein isothiocyanate in 250 μl of 100 mM bicine, pH 9.0 for 2 h at room temperature. Excess reagent was removed by repeated washing with TBS in a VivaSpin-2 centrifugal concentrator with a 10-kDa cutoff membrane (VIVAproducts). Liposomes were prepared by combining 3 μmole of distearoylphosphatidylcholine, 1.25 μmole of mixed porcine brain gangliosides (Avanti Polar Lipids), 1.75 μmole of cholesterol, and 0.25 μg of aminofluorescein coupled to distearoylphosphatidyl ethanolamine-polyethylene glycol 2000-N-hydroxysuccinimide (Cayman Chemicals). The mixture was dried, resuspended in 2 ml of TBS, sonicated for 1 min and extruded 5 times through 0.2 μm aluminum filters (Anitop).

### Array screening

All procedures were conducted in binding buffer containing 0.15 M NaCl, 25 mM Tris–Cl, pH 7.8, 2.5 mM CaCl_2_. Biotinylated receptor fragments were dissolved in binding buffer containing 0.1% bovine serum albumin. Test binding assays, using the ligands indicated in Table 2, were used to confirm that the wells were saturated with biotin-tagged CRDs (Fig. S34). Either clear or black streptavidin-coated 96-well plates (Thermo Fisher Scientific) were incubated overnight at 4 °C in duplicate with 60 μl aliquots of coating stocks, and the wells were washed three times with binding buffer. Labeled microorganisms suspended in binding buffer containing 0.1% bovine serum albumin were added in 60 μl aliquots. After incubation for 2 to 4 h at 4 °C, wells were washed 3 times with binding buffer and scanned directly on a Victor3 multiwell plate reader (PerkinElmer). Unlabeled microorganisms were bound in the same way. After three washes with binding buffer, 60 μl of 5 μg/ml Syto9 dye (Life Technologies) in binding buffer was added to each well and incubated for 15 min at room temperature. Wells were washed a further five times with binding buffer before reading. For counterstaining of PNAG with fluorescein-labeled wheat germ agglutinin, the initial binding incubation and washes were followed by a second incubation with 10 μg/ml lectin for 2 h at 4 °C, followed by three further washes with binding buffer. In all cases, averages of duplicate wells are plotted, with error bars representing the range of values. For each ligand used to screen the array, average percentage errors given in the legends were determined as the difference between the values for duplicate wells as a percentage of the average of the values. The overall average errors for each ligand were based on signals that were greater than 10% of the maximum signal. When samples were screened in multiple experiments, similar values normalized to the highest signal were obtained and a representative experiment is shown.

## Data availability

All data are contained in the manuscript and supporting information.

## Supporting information

This article contains supporting information.

## Conflict of interest

The authors declare that they have no conflicts of interest with the contents of this article.
