# Supplementary material for: A human lectin array for characterizing host-pathogen interactions
Source: J Biol Chem. 2024 Oct 9;300(11):107869. doi: 10.1016/j.jbc.2024.107869 (PMC11566865; doi:10.1016/j.jbc.2024.107869)
Supplement: Supporting information Figures S1-S34 [file mmc1.pdf]

Supporting Information

A human lectin array for characterizing host-pathogen interactions

Stefi V. Benjamin, Sabine A. F. Jégouzo, Chloe Lieng, Connor Daniels, Marine Coispeau, Rikin J. Lau, Suyeon Kim, Yasmine Metaxa, James Philpott, Tiannuo Li, Chao Dai, Xin Wang, Maddy L. Newby, Gerald B. Pier, Max Crispin, Abigail Clements, Maureen E. Taylor, and Kurt Drickamer

Figures S1-S33. Sequences of cDNAs for biotin-tagged CRDs [This file].

Figure S34. Saturation curves for coating of assay wells [This file].

Tables S1-S5. Binding data for Figures 2-6 [Excel file].

Sequences of cDNAs for biotin-tagged CRDs

Sequences of cloned CRDs are shown in black. Appended initiation sequences, biotin tags, and variations in the sequences compared to the genomic sequence in the National Center for Biotechnology Information database are indicated in red.

Figure S1. Mannose-binding protein / lectin (MBP)

```
                                   MetAlaValGlyAsnLysPhe
         ggatccgatcttggaggatgattaaatggccgttgggaacaagttc

PheLeuThrAsnGlyGluIleMetThrPheGluLysValLysAlaLeuCysValLysPhe
ttcctgaccaatggtgaaataatgacctttgaaaaagtgaaggccttgtgtgtcaagttc

GlnAlaSerValAlaThrProArgAsnAlaAlaGluAsnGlyAlaIleGlnAsnLeuIle
caggcctctgtggccacccccaggaatgctgcagagaatggagccattcagaatctcatc

LysGluGluAlaPheLeuGlyIleThrAspGluLysThrGluGlyGlnPheValAspLeu
aaggaggaagccttcctgggcatcactgatgagaagacagaagggcagtttgtggatctg

ThrGlyAsnArgLeuThrTyrThrAsnTrpAsnGluGlyGluProAsnAsnAlaGlySer
acaggaaatagactgacctacacaaactggaacgagggtgaacccaacaatgctggttct

AspGluAspCysValLeuLeuLeuLysAsnGlyGlnTrpAsnAspValProCysSerThr
gatgaagattgtgtattgctactgaaaaatggccagtggaatgacgtcccctgctccacc

SerHisLeuAlaValCysGluPheProIleLeuAsnAspIlePheGluAlaGlnLysIle
tcccatctggccgtctgtgagttccctatcctgaatgacatcttcgaagcacagaaaatc

GluTrpHisGlu***
gagtggcatgagtagaattc
```

Figure S2. Surfactant protein A (SP-A)

```
  ***MetAlaLeuAsnAspIlePheGluAlaGlnLysIleGluTrpHisGluGlySer
  taaatggccctgaatgacatcttcgaagcacagaaaatcgagtggcatgagggatcc

AlaHisLeuAspGluGluLeuGlnAlaThrLeuHisAspPheArgHisGlnIleLeuGln
gctcatctagatgaggagctccaagccacactccacgactttagacatcaaatcctgcag

ThrArgGlyAlaLeuSerLeuGlnGlySerIleMetThrValGlyGluLysValPheSer
acaaggggagccctcagtctgcagggctccataatgacagtaggagagaaggtcttctcc

SerAsnGlyGlnSerIleThrPheAspAlaIleGlnGluAlaCysAlaArgAlaGlyGly
agcaatgggcagtccatcacttttgatgccattcaggaggcatgtgccagagcaggcggc

ArgIleAlaValProArgAsnProGluGluAsnGluAlaIleAlaSerPheValLysLys
cgcattgctgtcccaaggaatccagaggaaaatgaggccattgcaagcttcgtgaagaag

TyrAsnThrTyrAlaTyrValGlyLeuThrGluGlyProSerProGlyAspPheArgTyr
tacaacacatatgcctatgtaggcctgactgagggtcccagccctggagacttccgctac

SerAspGlyThrProValAsnTyrThrAsnTrpTyrArgGlyGluProAlaGlyArgGly
tcagacgggacccctgtaaactacaccaactggtaccgaggggagcccgcaggtcgggga

LysGluGlnCysValGluMetTyrThrAspGlyGlnTrpAsnAspArgAsnCysLeuTyr
aaagagcagtgtgtggagatgtacacagatgggcagtggaatgacaggaactgcctgtac

SerArgLeuThrIleCysGluPhe***
tcccgactgaccatctgtgagttctgaattc
```

Figure S3. Surfactant protein D (SP-D)

```
                                                       MetAlaVal
                                 ggatccgatcttggaggatgattaaatggccgtc

GlyGluLysIlePheLysThrAlaGlyPheValLysProPheThrGluAlaGlnLeuLeu
ggggagaagatttcaagacagcaggctttgtaaaaccatttacggaggcacagctgctg

CysThrGlnAlaGlyGlyGlnLeuAlaSerProArgSerAlaAlaGluAsnAlaAlaLeu
tgcacacaggctggtggacagttggcctctccacgctctgccgctgagaatgccgccttg

GlnGlnLeuValValAlaLysAsnGluAlaAlaPheLeuSerMetThrAspSerLysThr
caacagctggtcgtagctaagaacgaggctgctttcctgagcatgactgattccaagaca

GluGlyLysPheThrTyrProThrGlyGluSerLeuValTyrSerAsnTrpAlaProGly
gagggcaagttcacctaccccacaggagagtccctggtctattccaactgggccccaggg

GluProAsnAspAspGlyGlySerGluAspCysValGluIlePheThrAsnGlyLysTrp
gagcccaacgatgatggcgggtcagaggactgtgtggagatcttcaccaatggcaagtgg

AsnAspArgAlaCysGlyGluLysArgLeuValValCysGluPheLeuAsnAspIlePhe
aatgacagggcttgtggagaaaagcgtcttgtggtctgcgagttcctgaatgacatcttc

GluAlaGlnLysIleGluTrpHisGlu***
gaagcacagaaaatcgagtggcatgagtgaattc
```

Figure S4. Collectin K1 (ColK1)

```
                                                  ggatccgatc

                     MetAlaThrGluSerLysIleTyrLeuLeuValLysGluGluLys
ttggaggatgattaaatggccacggagagcaagatctacctgctggtgaaggaggagaag

ArgTyrAlaAspAlaGlnLeuSerCysGlnGlyArgGlyGlyThrLeuSerMetProLys
cgctacgcggacgcccagctgtcctgccagggccgcgggggcacgctgagcatgcccaag

AspGluAlaAlaAsnGlyLeuMetAlaAlaTyrLeuAlaGlnAlaGlyLeuAlaArgVal
gacgaggctgccaatggcctgatggccgcatacctggcgcaagccggcctggcccgtgtc

PheIleGlyIleAsnAspLeuGluLysGluGlyAlaPheValTyrSerAspHisSerPro
ttcatcggcatcaacgacctggagaaggagggcgccttcgtgtactctgaccactccccc

MetArgThrPheAsnLysTrpArgSerGlyGluProAsnAsnAlaTyrAspGluGluAsp
atgcggaccttcaacaagtggcgcagcggtgagcccaacaatgcctacgacgaggaggac

CysValGluMetValAlaSerGlyGlyTrpAsnAspValAlaCysHisThrThrMetTyr
tgcgtggagatggtggcctcgggcggctggaacgacgtggcctgccacaccaccatgtac

PheMetCysGluPheAspLeuAsnAspIlePheGluAlaGlnLysIleGluTrpHisGlu
ttcatgtgtgagtttgacctgaatgacatcttcgaagcacagaaaatcgagtggcatgag

***
tagaattc
```

Figure S5. Langerin (Langerin)

```
                                                 ggatccgatcttggag

        MetAlaGlnValValSerGlnGlyTrpLysTyrPheLysGlyAsnPheTyr
gatgattaaatggcccaggtggtttctcaaggctggaagtacttcaaggggaacttctat

TyrPheSerLeuIleProLysThrTrpTyrSerAlaGluGlnPheCysValSerArgAsn
tacttttctctcattccaaagacctggtatagtgccgagcagttctgtgtgtccaggaat

SerHisLeuThrSerValThrSerGluSerGluGlnGluPheLeuTyrLysThrAlaGly
tcacacctgacctcggtgacctcagagagcgagcaggagtttctgtataaaacagcgggg

GlyLeuIleTyrTrpIleGlyLeuThrLysAlaGlyMetGluGlyAspTrpSerTrpVal
ggactcatctactggattggactgactaaagcagggatggaaggggactggtcctgggtg

AsnAspThrProPheAsnLysValGlnSerAlaArgPheTrpIleProGlyGluProAsn
aatgacacgccattcaacaaggtccaaagtgcgaggttctggattccaggtgagcccaac

AspAlaGlyAsnAsnGluHisCysGlyAsnIleLysAlaProSerLeuGlnAlaTrpAsn
gatgctgggaacaatgaacactgtggcaatataaaggctccctcacttcaggcctggaat

AspAlaProCysAspLysThrPheLeuPheIleCysLysArgProTyrValProSerGlu
gatgccccatgtgacaaaacgtttcttttcatttgtaagcgaccctatgtcccatcagaa


ProGlyLeuAsnAspIlePheGluAlaGlnLysIleGluTrpHisGlu***
ccgggactgaatgacatcttcgaagcacagaaaatcgagtggcatgagtagaagctt
```

Figure S6. DC-SIGN / CD209 (DC-SIGN)

```
                              MetAlaGluArgLeuCysHisProCysProTrp
  ggatccgatcttggaggatgattaaatggccgaacgcctgtgccacccctgtccctgg

GluTrpThrPhePheGlnGlyAsnCysTyrPheMetSerAsnSerGlnArgAsnTrpHis
gaatggacattcttccaaggaaactgttacttcatgtctaactcccagcggaactggcac

AspSerIleTheAlaCysLysGluValGlyAlaGlnLeuValValIleLysSerAlaGlu
gactccatcaccgcctgcaaagaagtgggggcccagctcgtcgtaatcaaaagtgctgag

GluGlnAsnPheLeuGlnLeuGlnSerSerArgSerAsnArgPheThrTrpMetGlyLeu
gagcagaacttcctacagctgcagtcttccagaagtaaccgcttcacctggatgggactt

SerAspLeuAsnGlnGluGlyThrTrpGlnTrpValAspGlySerProLeuLeuProSer
tcagatctaaatcaggaaggcacgtggcaatgggtggacggctcacctctgttgcccagc

PheLysGlyTyrTrpAsnArgGlyGluProAsnAsnValGlyGluGluAspCysAlaGlu
ttcaagcagtattggaacagaggagagcccaacaacgttggggaggaagactgcgcggaa

PheSerGlyAsnGlyTrpAsnGluGluLysCysAsnLeuAlaLysPheTrpIleCysLys
tttagtggcaatggctggaacgacgacaaatgtaatcttgccaaattctggatctgcaaa

LysSerAlaAlaSerCysSerArgAspGluGluGlnPheLeuSerProAlaProAlaThr
aagtccgcagcctcctgctccagggatgaagaacagtttctttctccagcccctgccacc

ProAsnProProProAlaGlyLeuAsnAspIlePheGluAlaGlnLysIleGluTrpHis
ccaaacccccctcctgcgggactgaatgacatcttcgaagcacagaaaatcgagtggcat

Glu***
gagtaggaattc
```

Figure S7. DC-SIGNR / L-SIGN / CD299 (DC-SIGNR)

```
                                                  ggatccgatcttggag

         MetAlaGluArgLeuCysArgHisCysProLysAspTrpThrPhePheGln
gatgattaaatggccgaacgcctgtgccgccactgtcccaaggactggacattcttccaa

GlyAsnCysTyrPheMetSerAsnSerGlnArgAsnTrpHisAspSerValThrAlaCys
ggaaactgttacttcatgtctaactcccagcggaactggcacgactccgtcaccgcctgc

GlnGluValArgAlaGlnLeuValValIleLysThrAlaGluGluGlnAsnPheLeuGln
caggaagtgagggcccagctcgtcgtaatcaaaactgctgaggagcagaacttcctacag

LeuGlnThrSerArgSerAsnArgPheSerTrpMetGlyLeuSerAspLeuAsnGlnGlu
ctgcagacttccaggagtaaccgcttctcctggatgggactttcagacctaaatcaggaa

GlyThrTrpGlnTrpValAspGlySerProLeuSerProSerPheGlnArgTyrTrpAsn
ggcacgtggcaatgggtggacggctcacctctgtcacccagcttccagcggtactggaac

SerGlyGluProAsnAsnSerGlyAsnGluAspCysAlaGluPheSerGlySerGlyTrp
agtggagaacccaacaatagcgggaatgaagactgtgcggaatttagtggcagtggctgg

AsnAspAsnArgCysAspValAspAsnTyrTrpIleCysLysLysProAlaAlaCysPhe
aacgacaatcgatgtgacgttgacaattactggatctgcaaaaagcccgcagcctgcttc

ArgAspGluGlyLeuAsnAspIlePheGluAlaGlnLysIleGluTrpHisGlu***
agagacgaaggactgaatgacatcttcgaagcacagaaaatcgagtggcatgagtaggaa

ttc
```

Figure S8. LSECtin (LSECtin)

```
                                                MetAlaAsnSerCysGluProC
             ggatccgatcttggaggatgattaaatggccaactcctgcgagccgt

ysProThrSerTrpLeuSerPheGluGlySerCysTyrPhePheSerValProLysThrT
gccccacgtcgtggctgtccttcgagggctcctgctactttttctctgtgccaaagacga

hrTrpAlaAlaAlaGlnAspHisCysAlaAspAlaSerAlaHisLeuValIleValGlyG
cgtgggcggcggcgcaggatcactgcgcagatgccagcgcgcacctggtgatcgttgggg

lyLeuAspGluGlnGlyPheLeuThrArgAsnThrArgGlyArgGlyTyrTrpLeuGlyL
gcctggatgagcagggcttcctcactcggaacacgcgtggccgtggttactggctgggcc

euArgAlaValArgHisLeuGlyLysValGlnGlyTyrGlnTrpValAspGlyValSerL
tgagggctgtgcgccatctgggcaaggttcagggctaccagtgggtggacggagtctctc

euSerPheSerHisTrpAsnGlnGlyGluProAsnAspAlaTrpGlyArgGluAsnCysV
tcagcttcagccactggaaccagggagagcccaatgacgcttgggggcgcgagaactgtg

alMetMetLeuHisThrGlyLeuTrpAsnAspAlaProCysAspSerGluLysAspGlyT
tcatgatgctgcacacggggctgtggaacgacgcaccgtgtgacagcgagaaggacggct

rpIleCysGluLysArgHisAsnCysGlyLeuAsnAspIlePheGluAlaGlnLysIleG
ggatctgtgagaaaaggcacaactgcggactgaatgacatcttcgaagcacagaaaatcg

luTrpHisGlu***
agtggcatgagtagaattc
```

Figure S9. Endo180 / UPARAP (Endo180 CRD 2)

```
                                    aaggatccgatcttggaggatga

   MetAlaValGluCysGluProSerTrpGlnProPheGlnGlyHisCysTyrArgLe
ttaaatggccgtggagtgcgagccgagctggcagcccttccagggccactgctaccgcct

uGlnAlaGluLysArgSerTrpGlnGluSerLysLysAlaCysLeuArgGlyGlyGlyAs
gcaggccgagaagcgcagctggcaggagtccaagaaggcatgtctacggggcggtggcga

pLeuValSerIleHisSerMetAlaGluLeuGluPheIleThrLysGlnIleLysGlnGl
cctggtcagcatccacagcatggcggagctggaattcatcaccaagcagatcaagcaaga

uValGluGluLeuTrpIleGlyLeuAsnAspLeuLysLeuGlnMetAsnPheGluTrpSe
ggtggaggagctgtggatcggcctcaacgatttgaaactgcagatgaattttgagtggtc

rAspGlySerLeuValSerPheThrHisTrpHisProPheGluProAsnAsnPheArgAs
tgacgggagccttgtgagcttcacccactggcacccctttgagcccaacaacttccggga

pSerLeuGluAspCysValThrIleTrpGlyProGluGlyArgTrpAsnAspSerProCy
cagtctggaggactgtgtcaccatctggggcccggaaggccgctggaacgacagtccctg

sAsnGlnSerLeuProSerIleCysLysLysGlyLeuAsnAspIlePheGluAlaGlnLy
taaccagtccttgccatccatctgcaagaagggactgaatgacatcttcgaagcacagaa

sIleGluTrpHisGlu***
aatcgagtggcatgagtagaagctt
```

Figure S10. Dectin-1 (Dectin-1)

```
                                  MetGlyValLeuSerSerProCys
              ggatccgatcttggaggatgattaaatggggttctttccagcccttgt

ProProAsnTrpIleIleTyrGluLysSerCysTyrLeuPheSerMetSerLeuAsnSer
cctcctaattggattatatatgagaagagctgttatctattcagcatgtcactaaattcc

TrpAspGlySerLysArgGlnCysTrpGlnLeuGlySerAsnLeuLeuLysIleAspSer
tgggatggaagtaaaagacaatgctggcaactgggctctaatctcctaaagatagacagc

SerAsnGluLeuGlyPheIleValLysGlnValSerSerGlnProAspAsnSerPheTrp
tcaaatgaattgggatttatagtaaaacaagtgtcttcccaacctgataattcattttgg

IleGlyLeuSerArgProGlnThrGluValProTrpLeuTrpGluAspGlySerThrPhe
ataggcctttctcggccccagactgaggtaccatggctctgggaggatggatcaacattc

SerSerAsnLeuPheGlnIleArgThrThrAlaThrGlnGluAsnProSerProAsnCys
tcttctaacttatttcagatcagaaccacagctacccaagaaaacccatctccaaattgt

ValTrpIleHisValSerValIleTyrAspGlnLeuCysSerValProSerTyrSerIle
gtatggattcacgtgtcagtcatttatgaccaactgtgtagtgtgccctcatatagtatt

CysGluLysLysPheSerMetLeuAsnAspIlePheGluAlaGlnLysIleGluTrpHis
tgtgagaagaagttttcaatgctgaatgacatcttcgaagcacagaaaatcgagtggcat

Glu***
gagtagaa
```

Figure S11. Asialoglycoprotein receptor 1 (ASGPR1)

```
                                       MetAlaLeuSerCysGlnMetAlaAl
        ggatccgatcttggaggatgattaaatggccctgagctgtcagatggcggc

aLeuGlnGlyAsnGlySerGluArgThrCysCysProValAsnTrpValGluHisGluAr
gctccagggcaatggctcagaaaggacctgctgcccggtcaactgggtggagcacgagcg

gSerCysTyrTrpPheSerArgSerGlyLysAlaTrpAlaAspAlaAspAsnTyrCysAr
cagctgctactggttctctcgctccgggaaggcctgggctgacgccgacaactactgccg

gLeuGluAspAlaHisLeuValValValThrSerTrpGluGluGlnLysPheValGlnHi
gctggaggacgcgcacctggtggtggtcacgtcctgggaggagcagaaatttgtccagca

sHisIleGlyProValAsnThrTrpMetGlyLeuHisAspGlnAsnGlyProTrpLysTr
ccacataggccctgtgaacacctggatgggcctccacgaccaaaacgggccctggaagtg

pValAspGlyThrAspTyrGluThrGlyPheLysAsnTrpArgProGluGlnProAspAs
ggtggacgggacggactacgagacgggcttcaagaactggaggccggagcagccggacga

pTrpTyrGlyHisGlyLeuGlyGlyGlyGluAspCysAlaHisPheThrAspAspGlyAr
ctggtacggccacgggctcggaggaggcgaggactgtgcccacttcaccgacgacggccg

gTrpAsnAspAspValCysGlnArgProTyrArgTrpValCysGluThrGluLeuAspLy
ctggaacgacgacgtctgccagaggccctaccgctgggtctgcgagacagagctggacaa

sAlaSerGlnGluProProLeuLeuGlyLeuAsnAspIlePheGluAlaGlnLysIleGl
ggccagccaggagccacctctccttggactgaatgacatcttcgaagcacagaaaatcga

uTrpHisGlu***
gtggcatgagtagaattc
```

Figure S12. Asialoglycoprotein receptor 2 (ASGPR2)

```
                                                      MetAlaVal
                       ggatccgatcttggaggatgattaaatggccgtg

AlaCysGlnMetGluLeuLeuHisSerAsnGlySerGlnArgThrCysCysProValAsn
gcctgccagatggagctcctccacagcaacggctcccaaaggacctgctgccccgtcaac

TrpValGluHisGlnGlySerCysTyrTrpPheSerHisSerGlyLysAlaTrpAlaGlu
tgggtggagcaccaaggcagctgctactggttctctcactccgggaaggcctgggctgag

AlaGluLysTyrCysGlnLeuGluAsnAlaHisLeuValValIleAsnSerTrpGluGlu
gcggagaagtactgccagctggagaacgcacacctggtggtcatcaactcctgggaggag

GlnLysPheIleValGlnHisThrAsnProPheAsnThrTrpIleGlyLeuThrAspSer
cagaaattcattgtacaacacacgaaccccttcaatacctggataggtctcacggacagt

AspGlySerTrpLysTrpValAspGlyThrAspTyrArgHisAsnTyrLysAsnTrpAla
gatggctcttggaaatgggtggatggcacagactataggcacaactacaagaactgggct

ValThrGlnProAspAsnTrpHisGlyHisGluLeuGlyGlySerGluAspCysValGlu
gtcactcagccagataattggcacgggcacgagctgggtggaagtgaagactgtgttgaa

ValGlnProAspGlyArgTrpAsnAspAspPheCysLeuGlnValTyrArgTrpValCys
gtccagccggatggccgctggaacgatgacttctgcctgcaggtgtaccgctgggtgtgt

GluLysArgArgAsnAlaThrGlyGluValAlaGlyLeuAsnAspIlePheGluAlaGln
gagaaaaggcggaatgccaccggcgaggtggccggactgaatgacatcttcgaagcacag

LysIleGluTrpHisGlu***
aaaatcgagtggcatgagtagaattc
```

Figure S13. Macrophage galactose receptor (MGL)

```
                                            ggatccgatc

                 MetAlaLeuThrCysGlnValAlaThrLeuAsnAsnAsnAlaSer
ttggaggatgattaaatggccctgacctgccaggtggctactctcaacaacaatgcctcc

ThrGluGlyThrCysCysProValAsnTrpValGluHisGlnAspSerCysTyrTrpPhe
actgaagggacctgctgccccgtcaactgggtggagcaccaagacagctgctactggttc

SerHisSerGlyMetSerTrpAlaGluAlaGluLysTyrCysGlnLeuLysAsnAlaHis
tctcactctgggatgtcctgggccgaggctgagaagtactgccagctgaagaacgcccac

LeuValValIleAsnSerArgGluGluGlnAsnPheValGlnLysTyrLeuGlySerAla
ctggtggtcatcaactccagggaggagcagaatttgtccagaaatatctaggctccgca

TyrThrTrpMetGlyLeuSerAspProGluGlyAlaTrpLysTrpValAspGlyThrAsp
tacacctggatgggcctcagtgaccctgaaggagcctggaagtgggtggatggaacagac

TyrAlaThrGlyPheGlnAsnTrpLysProGlyGlnProAspAspTrpGlnGlyHisGly
tatgcgaccggcttccagaactggaagccaggccagccagacgactggcaggggcacggg

LeuGlyGlyGlyGluAspCysAlaHisPheHisProAspGlyArgTrpAsnAspAspVal
ctgggtggaggcgaggactgtgctcacttccatccagacggcaggtggaatgacgacgtc

CysGlnArgProTyrHisTrpValCysGluAlaGlyLeuGlyGlnThrSerGlnGluSer
tgccagaggccctaccactgggtctgcgaggctggcctgggtcagaccagccaggagagt

HisGlyLeuAsnAspIlePheGluAlaGlnLysIleGluTrpHisGlu***
cacggactgaatgacatcttcgaagcacagaaaatcgagtggcatgagtagaattc
```

Figure S14. Galectin 1 (Galectin 1)

```
                                 MetAlaSerGlyLeuValAlaSerAsnLeuAsn
  ggatccgatcttggaggatgattaaatggcctcgggtctggtcgccagcaacctgaat

LeuLysProGlyGluSerLeuArgValArgGlyGluValAlaProAspAlaLysSerPhe
ctcaaacctggagagtctcttcgagtgcgaggcgaggtggctcctgacgctaagagcttc

ValLeuAsnLeuGlyLysAspSerAsnAsnLeuSerLeuHisPheAsnProArgPheAsn
gtgctgaacctgggcaaagactctaacaacctgtcactgcacttcaaccctcgcttcaac

AlaHisGlyAspAlaAsnThrIleValSerAsnSerLysAspGlyGlyAlaTrpGlyThr
gcccacggcgacgccaacaccatcgtgtctaactccaaggacggcggggcctgggggacc

GluGlnArgGluAlaValPheProPheGlnProGlySerValAlaGluValSerIleThr
gagcagcgggaggctgtctttcccttccagcctggaagtgttgcagaggtgtccatcacc

PheAspGlnAlaAsnLeuThrValLysLeuProAspGlyTyrGluPheLysPheProAsn
ttcgaccaggccaacctgaccgtcaagctgccagatggatacgagttcaagttccccaac

ArgLeuAsnLeuGluAlaIleAsnTyrMetAlaAlaAspGlyAspPheLysIleLysSer
cgcctcaacctggaggccatcaactacatggcagctgacggtgacttcaagatcaaatcg

ValAlaPheAspGlyLeuAsnAspIlePheGluAlaGlnLysIleGluTrpHisGlu***
gtggcctttgacggactgaatgacatcttcgaagcacagaaaatcgagtggcatgagtag

aattc
```

Figure S15. Galectin 2 (Galectin 2)

```
                                   MetThrGlyGluLeuGluValLysAsnMetAsp
  ggatccgatcttggaggatgattaaatgacgggggaacttgaggttaagaacatggac

MetLysProGlySerThrLeuLysIleThrGlySerIleAlaAspGlyThrAspGlyPhe
atgaagccggggtcaaccctgaagatcacaggcagcatcgccgatggcactgatggcttt

ValIleAsnLeuGlyGlnGlyThrAspLysLeuAsnLeuHisPheAsnProArgPheSer
gtaattaatctgggccaggggacagacaagctgaacctgcatttcaaccctcgcttcagc

GluSerThrIleValMetAsnSerLeuAspGlySerAsnTrpGlyGlnGluGlnArgGlu
gaatccaccattgtctctaactcattggacggcagcaactgggggcaagaacaacgggaa

AspHisLeuSerPheSerProGlySerGluValLysPheThrValThrPheGluSerAsp
gatcacctgtcgttcagcccagggtcagaggtcaagttcacagtgacctttgagagtgac

LysPheLysValLysLeuProAspGlyHisGluLeuThrPheProAsnArgLeuGlyHis
aaattcaaggtgaagctgccagatgggcacgagctgacttttcccaacaggctgggtcac

SerHisLeuSerTyrLeuSerValArgGlyGlyPheAsnMetSerSerPheLysLeuLys
agccacctgagctacctgagcgtaaggggcgggttcaacatgtcctctttcaagttaaaa

GluGlyLeuAsnAspIlePheGluAlaGlnLysIleGluTrpHisGlu***
gaaggactgaatgacatcttcgaagcacagaaaatcgagtggcatgagtagaattc
```

Figure S16. Galectin 3 (Galectin 3)

```
                                 MetGlyProLeuIleValProTyrAsnLeuProLeu
ggatccgatcttggaggatgattaaatggggccactgattgtgccttataacctgcctttg

ProGlyGlyValValProArgMetLeuIleThrIleLeuGlyThrValLysProAsnAla
cctgggggagtggtgcctcgcatgctgataacaattctgggcacggtgaagcccaatgca

AsnArgIleAlaLeuAspPheGlnArgGlyAsnAspValAlaPheHisPheAsnProArg
aacagaattgctttagatttccaaagagggaatgatgttgccttccactttaacccacgc

PheAsnGluAsnAsnArgArgValIleValSerAsnThrLysLeuAspAsnAsnTrpGly
ttcaatgagaacaacaggagagtcattgtttcaaatacaaagctggataataactgggga

ArgGluGluArgGlnSerValPheProPheGluSerGlyLysProPheLysIleGlnVal
agggaagaaagacagtcggttttcccatttgaaagtgggaaaccattcaaaatacaagta

LeuValGluProAspHisPheLysValAlaValAsnAspAlaHisLeuLeuGlnTyrAsn
ctggttgaacctgaccacttcaaggttgcagtgaatgatgctcacttgttgcagtacaat

HisArgValLysLysLeuAsnGluIleSerLysLeuGlyIleSerGlyAspIleAspLeu
catcgggttaaaaaactcaatgaaatcagcaaactgggaatttctggtgacatagacctc

ThrSerAlaSerTyrThrMetIleGlyLeuAsnAspIlePheGluAlaGlnLysIleGlu
accagtgcttcatataccatgataggactgaatgacatcttcgaagcacagaaaatcgag

TrpHisGlu***
tggcatgagtagaattc
```

Figure S17. Galectin 7 (Galectin 7)

```
                           MetAlaSerAsnValProHisLysSerSerLeuPr
ggatccgatcttggaggatgattaaatggcctccaacgtcccccacaagtcctcactgcc

oGluGlyIleArgProGlyThrValLeuArgIleArgGlyLeuValProProAsnAlaSe
cgagggcatccgccctggcacggtgctgcgtattcgcggcttggttcctcccaatgccag

rArgPheHisValAsnLeuLeuSerGlyGluGluGlnGlySerAspAlaAlaLeuHisPh
caggttccatgtaaacctgctgtcgggggaggagcagggctccgatgccgcgctgcattt

eAsnProArgLeuAspThrSerGluValValPheAsnSerLysGluGlnGlySerTrpGl
caacccccggctggacacgtcggaggtggtcttcaacagcaaggagcaaggctcctgggg

yArgGluGluArgGlyProGlyValProPheGlnArgGlyGlnProPheGluValLeuIl
ccgcgaggagcgcgggccgggcgttcctttccagcgcgggcagcccttcgaggtgctcat

eIleAlaSerAspAspGlyPheLysAlaValValGlyAspAlaGlnTyrHisHisPheAr
catcgcgtcagacgacggcttcaaggccgtggttggggacgcccagtaccaccacttccg

gHisArgLeuProLeuAlaArgValArgLeuValGluValGlyGlyAspValGlnLeuAs
ccaccgcctgccgctggcgcgcgtgcgcctggtggaggtgggcggggacgtgcagctgga

pSerValArgIlePheGlyLeuAsnAspIlePheGluAlaGlnLysIleGluTrpHisGl
ctccgtgaggatcttcggactgaatgacatcttcgaagcacagaaaatcgagtggcatga

u***
gtagaattc
```

Figure S18. Galectin 4 N-terminal CRD (Galectin 4N)

```
                                 MetAlaProTyrTyrGlnProIleProGlyGlyLe
ggatccgatcttggaggatgattaaatggccccttactaccagcccatcccgggcgggct

uAsnValGlyMetSerValTyrIleGlnGlyValAlaSerGluHisMetLysArgPhePh
caacgtgggaatgtctgtttacatccaaggagtggccagcgagcacatgaagcggttctt

eValAsnPheValValGlyGlnAspProGlySerAspValAlaPheHisPheAsnProAr
cgtgaactttgtggttgggcaggatccgggctcagacgtcgccttccacttcaatccgcg

gPheAspGlyTrpAspLysValValPheAsnThrLeuGlnGlyGlyLysTrpGlySerGl
gtttgacggctgggacaaggtggtcttcaacacgttgcagggcgggaagtggggcagcga

uGluArgLysArgSerMetProPheLysLysGlyAlaAlaPheGluLeuValPheIleVa
ggagaggaagaggagcatgcccttcaaaaagggtgccgcctttgagctggtcttcatagt

lLeuAlaGluHisTyrLysValValValAsnGlyAsnProPheTyrGluTyrGlyHisAr
cctggctgagcactacaaggtggtggtaaatggaaatcccttctatgagtacgggcaccg

gLeuProLeuGlnMetValThrHisLeuGlnValAspGlyAspLeuGlnLeuGlnSerIl
gcttcccctacagatggtcacccacctgcaagtggatggggatctgcaacttcaatcaat

eGlyLeuAsnAspIlePheGluAlaGlnLysIleGluTrpHisGlu***
cggactgaatgacatcttcgaagcacagaaaatcgagtggcatgagtaggaattc
```

Figure S19. Galectin 4 C-terminal CRD (Galectin 4C)

```
                                 MetAlaThrPheAsnProProValProTyrPheGl
ggatccgatcttggaggatgattaaatggctaccttcaacccgcctgtgccatatttcgg

yArgLeuGlnGlyGlyLeuThrAlaArgArgThrIleIleIleLysGlyTyrValProPr
gaggctgcaaggagggctcacagctcgaagaaccatcatcatcaagggctatgtgcctcc

oThrGlyLysSerPheAlaIleAsnPheLysValGlySerSerGlyAspIleAlaLeuHi
cacaggcaagagctttgctatcaacttcaaggtgggctcctcaggggacatagctctgca

sIleAsnProArgMetGlyAsnGlyThrValValArgAsnSerLeuLeuAsnGlySerTr
cattaatccccgcatgggcaacggtaccgtggtccggaacagccttctgaatggctcgtg

pGlySerGluGluLysLysIleThrHisAsnProPheGlyProGlyGlnPhePheAspLe
gggctccgaggagaagaagatcacccacaacccatttggtcccggacagttctttgatct

uSerIleArgSerGlyLeuAspArgPheLysValTyrAlaAsnGlyGlnHisLeuPheAs
gtccattcgctctggcttggatcgcttcaaggtttacgccaatggccagcacctctttga

pPheAlaHisArgLeuSerAlaPheGlnArgValAspThrLeuGluIleGlnGlyAspVa
ctttgcccatcgcctctcggccttccagagggtggacacattggaaatccagggtgatgt

lThrLeuSerTyrValGlnIleGlyLeuAsnAspIlePheGluAlaGlnLysIleGluTr
caccttgtcctatgtccagatcggactgaatgacatcttcgaagcacagaaaatcgagtg

pHisGlu***
gcatgagtaggaattc
```

Figure S20. Galectin 8 C-terminal CRD (Galectin 8C)

```
                                 MetAlaGlnLeuArgLeuProPheAlaAlaArgLe
ggatccgatcttggaggatgattaaatggctcagcttaggctgccattcgctgcaaggtt

uAsnThrProMetGlyProGlyArgThrValValValLysGlyGluValAsnAlaAsnAl
gaacacccccatgggccctggacgaactgtcgtcgttaaaggagaagtgaatgcaaatgc

aLysSerPheAsnValAspLeuLeuAlaGlyLysSerLysAspIleAlaLeuHisLeuAs
caaaagctttaatgttgacctactagcaggaaaatcaaaggatattgctctacacttgaa

nProArgLeuAsnIleLysAlaPheValArgAsnSerPheLeuGlnGluSerTrpGlyGl
cccacgcctgaatattaaagcatttgtaagaaattcttttcttcaggagtcctggggaga

uGluGluArgAsnIleThrSerPheProPheSerProGlyMetTyrPheGluMetIleIl
agaagagagaaatattacctctttcccatttagtcctgggatgtactttgagatgataat

eTyrSerAspValArgGluPheLysValAlaValAsnGlyValHisSerLeuGluTyrLy
ttattcggatgttagagagttcaaggttgcagtaaatggcgtacacagcctggagtacaa

sHisArgPheLysGluLeuSerSerIleAspThrLeuGluIleAsnGlyAspIleHisLe
acacagatttaaagagctcagcagtattgacacgctggaaattaatggagacatccactt

uLeuGluValArgSerTrpGlyLeuAsnAspIlePheGluAlaGlnLysIleGluTrpHi
actggaagtaaggagctggggactgaatgacatcttcgaagcacagaaaatcgagtggca

sGlu***
tgagtagaattc
```

Figure S21. Galectin 9 N-terminal CRD (Galectin 9N)

```
                                   MetAlaSerGlnAlaProTyrLeuSerProAla
  ggatccgatcttggaggatgattaaatggcttcccaggctccctacctgagtccagct

ValProPheSerGlyThrIleGlnGlyGlyLeuGlnAspGlyLeuGlnIleThrValAsn
gtccccttttctgggactattcaaggaggtctccaggacggacttcagatcactgtcaat

GlyThrValLeuSerSerSerGlyThrArgPheAlaValAsnPheGlnThrGlyPheSer
gggaccgttctcagctccagtggaaccaggtttgctgtgaactttcagactggcttcagt

GlyAsnAspIleAlaPheHisPheAsnProArgPheGluAspGlyGlyTyrValValSer
ggaaatgacattgccttccacttcaaccctcggtttgaagatggagggtacgtggtgtcg

AsnThrArgGlnAsnGlySerTrpGlyProGluGluArgLysThrHisMetProPheGln
aacacgaggcagaacggaagctgggggcccgaggagaggaagacacacatgcctttccag

LysGlyMetProPheAspLeuSerPheLeuValGlnSerSerAspPheLysValMetVal
aaggggatgccctttgacctctccttcctggtgcagagctcagatttcaaggtgatggtg

AsnGlyIleLeuPheValGlnTyrPheHisArgValProPheHisArgValAspThrIle
aacggtatcctcttcgtgcagtacttccaccgcgtgcccttccaccgtgtggacaccatc

SerValAsnGlySerValGlnLeuSerTyrIleSerPheGlnGlyLeuAsnAspIlePhe
tccgtcaatggctctgtgcagctgtcctacatcagcttccagggactgaatgacatcttc

GluAlaGlnLysIleGluTrpHisGlu***
gaagcacagaaaatcgagtggcatgagtaggaattc
```

Figure S22. Galectin 9 C-terminal CRD (Galectin 9C)

```
                                 MetThrProAlaIleProProMetMetTyrProHi
ggatccgatcttggaggatgattaaatgactcccgccatcccacctatgatgtacccca

sProAlaTyrProMetProPheIleThrThrIleLeuGlyGlyLeuTyrProSerLysSe
ccccgcctatccgatgcctttcatcaccaccattctgggagggctgtacccatccaagtc

rIleLeuLeuSerGlyThrValLeuProSerAlaGlnArgPheHisIleAsnLeuCysSe
catcctcctgtcaggcactgtcctgcccagtgctcagaggttccacatcaacctgtgctc

rGlyAsnHisIleAlaPheHisLeuAsnProArgPheAspGluAsnAlaValValArgAs
tgggaaccacatcgccttccacctgaacccccgttttgatgagaatgctgtggtccgcaa

nThrGlnIleAspAsnSerTrpGlySerGluGluArgSerLeuProArgLysMetProPh
cacccagatcgacaactcctgggggtctgaggagcgaagtctgccccgaaaaatgccctt

eValArgGlyGlnSerPheSerValTrpIleLeuSerGluAlaHisSerLeuLysValAl
cgtccgtggccagagcttctcagtgtggatcttgtccgaagctcactcgctcaaggtggc

aValAspGlyGlnHisLeuPheGluTyrTyrHisArgLeuArgAsnLeuProThrIleAs
cgtggatggtcagcacctgtttgaatactaccatcgcctgaggaacctgcccaccatcaa

nArgLeuGluValGlyGlyAspIleGlnLeuThrHisValGlnThrGlyLeuAsnAspIl
cagactggaagtgggggcgacatccagctgacccatgtgcagacaggactgaatgacat

ePheGluAlaGlnLysIleGluTrpHisGlu***
cttcgaagcacagaaaatcgagtggcatgagtagaattc
```

Figure S23. Sialoadhesin (Siglec 1)

```
                                MetAlaSerTrpGlyValSerSerProGlnAsp
  ggatccgatcttggaggatgattaaatggcaagctggggtgttagcagtccgcaggat

ValGlnGlyValLysGlySerAlaLeuLeuIleProCysIlePheSerPheProAlaAsp
gttcagggtgttaaaggtagcgcactgctgattccgtgtattttagctttccggcagat

ValGluValProAspGlyIleThrAlaIleTrpTyrTyrAspTyrSerGlyGlnArgGln
gttgaagttccggatggtattaccgcaatctggtattatgattattcaggtcagcgtcag

ValValSerHisSerAlaAspProLysLeuValGluAlaArgPheArgGlyArgThrGlu
gttgttagccatagcgcagatccgaaactggttgaagcacgttttcgtggtcgtaccgaa

PheMetGlyAsnProGluHisArgValCysAsnLeuLeuLeuLysAspLeuGlnProGlu
tttatgggtaatccggaacatcgtgtttgtaatctgctgctgaaagatctgcagccggaa

AspSerGlySerTyrAsnPheArgPheGluIleSerGluValAsnArgTrpSerAspVal
gatagcggtagctataactttcgttttgaaatcagcgaagtgaaccgttggagtgatgtt

LysGlyThrLeuValThrValThrGluAlaLeuAsnAspIlePheGluAlaGlnLysIle
aaaggcaccctggttaccgttaccgaagcactgaatgacatcttcgaagcacagaaaatc

GluTrpHisGlu***
gagtggcatgagtagaattc
```

Figure S24. CD33 (Siglec 3)

```
                                    ggatccgatcttggaggatgattaa

MetAlaAspProAsnPheTrpLeuGlnValGlnGluSerValThrValGlnGluGlyLeu
atggccgatccgaattttggctgcaggttcaagaaagcgttaccgttcaagaaggtctg

AlaValLeuValProCysThrPhePheHisProIleProTyrTyrAspLysAsnSerPro
gcagttctggttccgtgtaccttttttcatccgattccgtattacgacaaaaacagtccg

ValHisGlyTyrTrpPheArgGluGlyAlaIleIleSerArgAspSerProValAlaThr
gttcatggttattggtttcgtgaaggtgcaattattagccgtgatagtccggttgccacc

AsnLysLeuAspGlnGluValGlnGluGluThrGlnGlyArgPheArgLeuLeuGlyAsp
aataaactggatcaagaagtgcaagaagaaacccagggtcgttttcgtctgctgggtgat

ProSerArgAsnAsnCysSerLeuSerIleValAspAlaArgArgArgAspAsnGlySer
ccgagccgtaataattgtagcctgagcattgttgatgcacgtcgtcgtgataatggtagc

TyrPhePheArgMetGluArgGlySerThrLysTyrSerTyrLysSerProGlnLeuSer
tatttttccgtatggaacgtggcagcaccaaatatagttataaaagtccgcagctgagc

ValHisValThrAspAlaLeuAsnAspIlePheGluAlaGlnLysIleGluTrpHisGlu
gttcatgttaccgatgcactgaatgacatcttcgaagcacagaaaatcgagtggcatgag

***
tagaattc
```

Figure S25. Siglec 5 (Siglec 5)

```
                                    MetAlaGluLysProValTyrGluLeuGlnVal
  ggatccgatcttggaggatgattaaatggcagaaaaaccggtgtatgaactgcaggtt

GlnLysSerValThrValGlnGluGlyLeuAlaValLeuValProCysSerPheSerTyr
cagaaaagcgttaccgttcaagaaggtctggcagttctggttccgtgtagctttagctat

ProTrpArgSerTrpTyrSerSerProProLeuTyrValTyrTrpPheArgAspGlyGlu
ccgtggcgtagctggtatagcagccctccgctgtatgtttattggtttcgtgatggtgaa

IleProTyrTyrAlaGluValValAlaThrAsnAsnProAspArgArgValLysProGlu
atcccgtattatgcagaagttgttgcaaccaataatccggatcgtcgtgttaaaccggaa

ThrGlnGlyArgPheArgLeuLeuGlyAspValGlnLysLysAsnCysSerLeuSerIle
acacagggtcgttttcgtctgctgggtgatgtgcagaaaaaaaactgtagcctgagcatt

GlyAspAlaArgMetGluAspThrGlySerTyrPhePheArgValGluArgGlyArgAsp
ggtgatgcacgtatggaagataccggtagctatttttttccgtgttgaacgtggccgtgat

ValLysTyrSerTyrGlnGlnAsnLysLeuAsnLeuGluValThrAlaLeuAsnAspIle
gtgaagtatagctatcagcagaataaactgaatctggaagttaccgcactgaatgacatc

PheGluAlaGlnLysIleGluTrpHisGlu***
ttcgaagcacagaaaatcgagtggcatgagtagaattc
```

Figure S26. Siglec 7 (Siglec 7)

```
                                    MetAlaGlnLysSerAsnArgLysAspTyrSer
  ggatccgatcttggaggatgattaaatggcccagaaaagcaatcgcaaagattattca

LeuThrMetGlnSerSerValThrValGlnGluGlyMetAlaValHisValArgCysSer
ctgaccatgcagagcagcgttaccgttcaagagggtatggcagttcatgttcgttgtagc

PheSerTyrProValAspSerGlnThrAspSerAspProValHisGlyTyrTrpPheArg
tttagctatccggttgatagccagaccgatagcgatccggttcatggttattggtttcgt

AlaGlyAsnAspIleSerTrpLysAlaProValAlaThrAsnAsnProAlaTrpAlaVal
gcaggtaatgatattagctggaaagcaccggttgccaccaataatccggcatgggcagtt

GlnGluGluThrArgAspArgPheHisLeuLeuGlyAspProGlnThrLysAsnCysThr
caagaagaaacacgcgatcgttttcatctgctgggtgatccgcagaccaaaaattgtacc

LeuSerIleArgAspAlaArgMetSerAspAlaGlyArgTyrPhePheArgMetGluLys
ctgagcattcgtgatgcacgtatgagtgatgcaggtcgttatttctttcgtatggaaaaa

GlyAsnIleLysTrpAsnTyrLysTyrAspGlnLeuSerValAsnValThrAlaLeuAsn
ggcaacatcaaatggaactacaaatatgatcagctgagcgttaatgttaccgccctgaat

AspIlePheGluAlaGlnLysIleGluTrpHisGlu***
gacatcttcgaagcacagaaaatcgagtggcatgagtagaattc
```

Figure S27. Siglec 9 (Siglec 9)

```
                                    MetAlaGlnThrSerLysLeuLeuThrMetGln
  ggatccgatcttggaggatgattaaatggcacagaccagcaaactgctgaccatgcag

SerSerValThrValGlnGluGlyLeuAlaValHisValProCysSerPheSerTyrPro
agcagcgttaccgttcaagagggtttagcagttcatgttccgtgtagctttagctatccg

SerHisGlyTrpIleTyrProGlyProValValHisGlyTyrTrpPheArgGluGlyAla
agccatggttggatttatccgggtcctgttgttcatggttattggtttcgtgaaggtgca

AsnThrAspGlnAspAlaProValAlaThrAsnAsnProAlaArgAlaValTrpGluGlu
aataccgatcaggatgcaccggttgccaccaataatccggcacgtgcagtttgggaagaa

ThrArgAspArgPheHisLeuLeuGlyAspProHisThrLysAsnCysThrLeuSerIle
acccgtgatcgttttcatctgctgggtgatccgcataccaaaaattgtaccctgagcatt

ArgAspAlaArgArgSerAspAlaGlyArgTyrPhePheArgMetGluLysGlySerIle
cgtgatgcacgtcgttcagatgcaggtcgttatttctttcgtatggaaaaaggcagcatc

LysTrpAsnTyrLysHisHisArgLeuSerValAsnValThrAlaLeuAsnAspIlePhe
aaatggaactataaacatcatcgtctgagcgttaatgttaccgcactgaatgacatcttc

GluAlaGlnLysIleGluTrpHisGlu***
gaagcacagaaaatcgagtggcatgagtagaattc
```

Figure S28. Siglec 11 (Siglec 11)

```
                                   ggatccgatcttggaggatgattaa

MetLeuAsnLysAspProSerTyrSerLeuGlnValGlnArgGlnValProValProGlu
atgctgaataaagatccgagctatagcctgcaggttcagcgtcaggttccggttccggaa

GlyLeuAlaValIleValSerCysAsnLeuSerTyrProArgAspGlyTrpAspGluSer
ggtctggcagttattgttagctgtaatctgagctatccgcgtgatggttgggatgaaagc

ThrAlaAlaTyrGlyTyrTrpPheLysGlyArgThrSerProLysThrGlyAlaProVal
accgcagcatatggttattggtttaaaggtcgtaccagtccgaaaacaggtgcaccggtt

AlaThrAsnAsnGlnSerArgGluValGluMetSerThrArgAspArgPheGlnLeuThr
gccaccaataatcagagccgtgaagttgaaatgagcacccgtgatcgttttcagctgacc

GlyAspProGlyLysGlySerCysSerLeuValIleArgAspAlaGlnArgGluAspGlu
ggtgatcctggtaaaggtagctgtagcctggttattcgtgatgcacagcgtgaagatgaa

AlaTrpTyrPhePheArgValGluArgGlySerArgValArgHisSerPheLeuSerAsn
gcatggtatttttttccgtgttgaacgtggtagccgtgttcgtcatagctttctgagcaat

AlaPhePheLeuLysValThrAlaLeuAsnAspIlePheGluAlaGlnLysIleGluTrp
gcatttttctgaaagttaccgccctgaatgacatcttcgaagcacagaaaatcgagtgg

HisGlu***
catgagtagaattc
```

Figure S29. Intelectin 1 (Intelectin 1)

```
                                                    MetAlaLeu
                                                  taaatggccctg

AsnAspIlePheGluAlaGlnLysIleGluTrpHisGluGlySerProSerLeuProArg
aatgacatcttcgaagcacagaaaatcgagtggcatgagggatccccatctctgcccaga

SerCysLysGluIleLysAspGluSerProSerAlaPheAspGlyLeuTyrPheLeuArg
agctgcaaggaaatcaaagacgaaagccctagtgcatttgatggcctgtattttctccgc

ThrGluAsnGlyValIleTyrGlnThrPheCysAspMetThrSerGlyGlyGlyGlyTrp
actgagaatggtgttatctaccagaccttctgtgacatgacctctgggggtggcggctgg

ThrLeuValAlaSerValHisGluAsnAspMetArgGlyLysCysThrValGlyAspArg
accctggtggccagcgtgcacgagaatgacatgcgtgggaagtgcacggtgggcgatcgc

TrpSerSerGlnGlnGlySerLysAlaValTyrProGluGlyAspGlyAsnTrpAlaAsn
tggtccagtcagcagggcagcaaagcagtctacccagagggggacggcaactgggccaac

TyrAsnThrPheGlySerAlaGluAlaAlaThrSerAspAspTyrLysAsnProGlyTyr
tacaacacctttggatctgcagaggcggccacgagcgatgactacaagaaccctggctac

TyrAspIleGlnAlaLysAspLeuGlyIleTrpHisValProAsnLysSerProMetGln
tacgacatccaggccaaggacctgggcatctggcacgtgcccaataagtcccccatgcag

HisTrpArgAsnSerSerLeuLeuArgTyrArgThrAspThrGlyPheLeuGlnThrLeu
cactggagaaacagctccctgctgaggtaccgcacggacactggcttcctccagacactg

GlyHisAsnLeuPheGlyIleTyrGlnLysTyrProValLysTyrGlyGluGlyLysCys
ggacataatctgtttggcatctaccagaaatatccagtgaaatatggagaaggaaagtgt

TrpThrAspAsnGlyProValIleProValValTyrAspPheGlyAspAlaGlnLysThr
tggactgacaacggcccggtgatccctgtggtctatgattttggcgacgcccagaaaaca

AlaSerTyrTyrSerProTyrGlyGlnArgGluPheThrAlaGlyPheValGlnPheArg
gcatcttattactcaccctatggccagcgggagttcactgcgggatttgttcagttcagg

ValPheAsnAsnGluArgAlaAlaAsnAlaLeuCysAlaGlyMetArgValThrGlyCys
gtatttaataacgagagagcagccaacgccttgtgtgctggaatgagggtcaccggatgt

AsnThrGluHisHisCysIleGlyGlyGlyGlyTyrPheProGluAlaSerProGlnGln
aacactgagcaccactgcattggtggaggaggatactttccagaggccagtccccagcag

CysGlyAspPheSerGlyPheAspTrpSerGlyTyrGlyThrHisValGlyTyrSerSer
tgtggagatttttctggttttgattggagtggatatggaactcatgttggttacagcagc

SerArgGluIleThrGluAlaAlaValLeuLeuPheTyrArg***
agccgtgagataactgaggcagctgtgcttctattctatcgttgaattc
```

Figure S30. Intelectin 2 (Intelectin 2)

```
        MetAlaLeuAsnAspIlePheGluAlaGlnLysIleGluTrpHisGluGly
      taaatggccctgaatgacatcttcgaagcacagaaaatcgagtggcatgaggga

SerPheSerSerLeuProArgSerCysLysGluIleLysGluArgSerHisSerAlaGly
tcctttcttccctgcctagaagctgcaaagaaatcaaggaacgcagccatagtgcaggt

AspGlyLeuTyrPheLeuArgThrLysAsnGlyValValTyrGlnThrPheCysAspMet
gatggcctgtattttctccgcaccaagaatggtgttgtctaccagaccttctgtgacatg

ThrSerGlyGlyGlyGlyTrpThrLeuValAlaSerValHisGluAsnAspMetArgGly
acttctgggggtggcggctggaccctggtggccagcgtgcacgagaatgacatgcgtggg

LysCysThrValGlyAspArgTrpSerSerGlnGlnGlyAsnLysAlaAspTyrProGlu
aagtgcacggtgggtgatcgctggtccagtcagcagggcaacaaagcagactacccagag

GlyAspGlyAsnTrpAlaAsnTyrAsnThrPheGlySerAlaGluAlaAlaThrSerAsp
ggggatggcaactgggccaactacaacacctttggatctgcagaggcggccacgagcgat

AspTyrLysAsnProGlyTyrTyrAspIleGlnAlaLysAspLeuGlyIleTrpHisVal
gactacaagaaccctggctactacgacatccaggccaaggacctgggcatctggcatgtg

ProAsnLysSerProMetGlnHisTrpArgAsnSerAlaLeuLeuArgTyrArgThrAsn
cccaacaagtcccccatgcagcattggagaaacagcgccctgctgaggtaccgcaccaac

ThrGlyPheLeuGlnArgLeuGlyHisAsnLeuPheGlyIleTyrGlnLysTyrProVal
actggcttcctccagagactgggacataatctgtttggcatctaccagaaatacccagtg

LysTyrArgSerGlyLysCysTrpAsnAspAsnGlyProAlaIleProValValTyrAsp
aaatacagatcagggaaatgttggaatgacaatggcccagccatacctgtggtctatgac

PheGlyAspAlaLysLysThrAlaSerTyrTyrSerProTyrGlyGlnArgGluPheVal
tttggtgatgctaagaagactgcatcttattactcaccgtatggtcaacgggaatttgtt

AlaGlyPheValGlnPheArgValPheAsnAsnGluArgAlaAlaAsnAlaLeuCysAla
gcaggattcgttcagttccgggtgtttaataacgagagagcagccaacgccctttgtgct

GlyIleLysValThrGlyCysAsnThrGluHisHisCysIleGlyGlyGlyGlyPhePhe
gggataaaagttactggctgtaacactgagcatcactgcatcggtggaggagggttcttc

ProGlnGlyLysProArgGlnCysGlyAspPheSerAlaPheAspTrpAspGlyTyrGly
ccacagggcaaaccccgtcagtgtggggacttctccgcctttgactgggatggatatgga

ThrHisValLysSerSerSerSerArgGluIleThrGluAlaAlaValLeuLeuPheTyr
actcacgttaagagcagcagcagtcgggagataacggaggcggctgtactcttgttctat

Arg***
agatgaattc
```

Figure S31. Mannose receptor R-type CRD (MMR-R)

```
                        Met
attacggattcactggaactctagataacgagggcaaaaatg

LysLysThrAlaIleAlaIlaAlaValAlaLeuAlaGlyPheAlaThrValAlaGlnAla
aaaaagacagctatcgcgattgcagtggcactggctggtttcgctaccgtagcgcaggcc

LeuLeuAspThrArgGlnPheLeuIleTyrAsnGluAspHisLysArgCysValAspAla
ctcctggacaccaggcaatttttaatctataatgaagatcacaagcgctgcgtggatgca

ValSerProSerAlaValGlnThrAlaAlaCysAsnGlnAspAlaGluSerGlnLysPhe
gtgagtcccagtgccgtccaaaccgcagcttgcaaccaggatgccgaatcacagaaattc

ArgTrpValSerGluSerGlnIleMetSerValAlaPheLysLeuCysLeuGlyValPro
cgatgggtgtccgaatctcagattatgagtgttgcatttaaattatgcctgggagtgcca

SerLysThrAspTrpValAlaIleThrLeuTyrAlaCysAspSerLysSerGluPheGln
tcaaaaacggactgggttgctatcactctctatgcctgtgactcaaaaagtgaatttcag

LysTrpGluCysLysAsnAspThrLeuLeuGlyIleLysGlyGluAspLeuPhePheAsn
aaatgggagtgcaaaaatgacacacttttggggatcaaaggagaagatttatttttttaac

TyrGlyAsnArgGlnGluLysAsnIleMetLeuTyrLysGlySerGlyLeuTrpSerArg
tacggcaacagacaagaaaagaatattatgctctacaagggatcgggtttatggagcagg

TrpLysIleTyrGlyThrThrAspAsnLeuCysSerArgGlyTyrGluAlaLeuAsnAsp
tggaagatctatggaaccacagacaatctgtgctccagaggttatgaagccctgaatgac

IlePheGluAlaGlnLysIleGluTrpHisGlu***
atcttcgaagcacagaaaatcgagtggcatgagtaggaattc
```

Figure S32. Ficolin 1 / Ficolin M

```
   MetAlaLeuAsnAspIlePheGluAlaGlnLysIleGluTrpHisGluGlySerCys
taaatggccctgaatgacatcttcgaagcacagaaaatcgagtggcatgagggatcctgt

AlaThrGlyProArgAsnCysLysAspLeuLeuAspArgGlyTyrPheLeuSerGlyTrp
gcgacaggcccacgcaactgcaaggacctgctagaccgggggtatttcctgagcggctgg

HisThrIleTyrLeuProAspCysArgProLeuThrValLeuCysAspMetAspThrAsp
cacaccatctacctgcccgactgccggcccctgactgtgctctgtgacatggacacggac

GlyGlyGlyTrpThrValPheGlnArgArgMetAspGlySerValAspPheTyrArgAsp
ggagggggctggaccgttttccagcggaggatggatggctctgtggacttctatcgggac

TrpAlaAlaTyrLysGlnGlyPheGlySerGlnLeuGlyGluPheTrpLeuGlyAsnAsp
tgggccgcatacaagcagggcttcggcagtcagctgggggagttctggctggggaatgac

AsnIleHisAlaLeuThrAlaGlnGlySerSerGluLeuArgValAspLeuValAspPhe
aacatccacgccctgactgcccagggaagcagcgagctccgtgtagacctggtggacttt

GluGlyAsnHisGlnPheAlaLysTyrLysSerPheLysValAlaAspGluAlaGluLys
gagggcaaccaccagtttgctaagtacaaatcattcaaggtggctgacgaggcagagaag

TyrLysLeuValLeuGlyAlaPheValGlyGlySerAlaGlyAsnSerLeuThrGlyHis
tacaagctggtactgggagcctttgtcgggggcagtgcgggtaattctctaacgggccac

AsnAsnAsnPhePheSerThrLysAspGlnAspAsnAspValSerSerSerAsnCysAla
aacaacaacttcttctccaccaaagaccaagacaatgatgtgagttcttcgaattgtgct

GluLysPheGlnGlyAlaTrpTrpTyrAlaAspCysHisAlaSerAsnLeuAsnGlyLeu
gagaagttccaaggagcctggtggtacgccgactgtcatgcttcaaacctcaatggtctc

TyrLeuMetGlyProHisGluSerTyrAlaAsnGlyIleAsnTrpSerAlaAlaLysGly
tacctcatgggaccccatgagagctatgccaatggtatcaactggagtgcggcgaagggg

TyrLysTyrSerTyrLysValSerGluMetLysValArgProAla***
tacaaatatagctacaaggtgtcagagatgaaggtgcggcccgcctagaattc
```

Figure S33. Chitinase 3-like lectin 2 / YKL39 (Chl3-L2)

```
          MetAlaLeuAsnAspIlePheGluAlaGlnLysIleGluTrpHisGlu
        taaatggccctgaatgacatcttcgaagcacagaaaatcgagtggcatgag

GlySerTyrLysLeuValCysTyrPheThrAsnTrpSerGlnAspArgGlnGluProGly
ggatcctacaaactggtttgctactttaccaactggtcccaggaccggcaggaaccagga

LysPheThrProGluAsnIleAspProPheLeuCysSerHisLeuIleTyrSerPheAla
aaattcacccctgagaatattgacccttcctatgctctcatctcatctattcattcgcc

SerIleGluAsnAsnLysValIleIleLysAspLysSerGluValMetLeuTyrGlnThr
agcatcgaaaacaacaaggttatcatcaaggacaagagtgaagtgatgctctaccagacc

IleAsnSerLeuLysThrLysAsnProLysLeuLysIleLeuLeuSerIleGlyGlyTyr
atcaacagtctcaaaaccaagaatcccaaactgaaaattctcttgtccattggagggtac

LeuPheGlySerLysGlyPheHisProMetValAspSerSerThrSerArgLeuGluPhe
ctgtttggttccaaagggttccaccctatggtggattcttctacatcacgcttggagttc

IleAsnSerIleIleLeuPheLeuArgAsnHisAsnPheAspGlyLeuAspValSerTrp
attaactccataatcctgtttctgaggaaccataactttgatggactggatgtaagctgg

IleTyrProAspGlnLysGluAsnThrHisPheThrValLeuIleHisGluLeuAlaGlu
atctacccagatcagaaagaaaacactcatttcactgtgctgattcatgagttagcagaa

AlaPheGlnLysAspPheThrLysSerThrLysGluArgLeuLeuLeuThrAlaGlyVal
gcctttcagaaggacttcacaaaatccaccaaggaaaggcttctcttgactgcgggcgta

SerAlaGlyArgGlnMetIleAspAsnSerTyrGlnValGluLysLeuAlaLysAspLeu
tctgcagggaggcaaatgattgataacagctatcaagttgagaaactggcaaaagatctg

AspPheIleAsnLeuLeuSerPheAspPheHisGlySerTrpGluLysProLeuIleThr
gatttcatcaacctcctgtcctttgacttccatgggtcttgggaaaagccccttatcact

GlyHisAsnSerProLeuSerLysGlyTrpGlnAspArgGlyProSerSerTyrTyrAsn
ggccacaacagccctctgagcaaggggtggcaggacagagggccaagctcctactacaat

ValGluTyrAlaValGlyTyrTrpIleHisLysGlyMetProSerGluLysValValMet
gtggaatatgctgtggggtactggatacataagggaatgccatcagagaaggtggtcatg

GlyIleProThrTyrGlyHisSerPheThrLeuAlaSerAlaGluThrThrValGlyAla
ggcatccccacatatgggcactccttcacactggcctctgcagaaaccaccgtgggggcc

ProAlaSerGlyProGlyAlaAlaGlyProIleThrGluSerSerGlyPheLeuAlaTyr
cctgcctctggccctggagctgctggacccatcacagagtcttcaggcttcctggcctat

TyrGluIleCysGlnPheLeuLysGlyAlaLysIleThrArgLeuGlnAspGlnGlnVal
tatgagatctgccagttcctgaaaggagccaagatcacgcggctccaggatcagcaggtt

ProTyrAlaValLysGlyAsnGlnTrpValGlyTyrAspAspValLysSerMetGluThr
ccctacgcagtcaaggggaaccagtgggtgggctatgatgatgtgaagagtatggagacc

LysValGlnPheLeuLysAsnLeuAsnLeuGlyGlyAlaMetIleTrpSerIleAspMet
aaggttcagttcttaaagaatttaaacctgggaggagccatgatctggtctattgacatg

AspAspPheThrGlyLysSerCysAsnGlnGlyProTyrProLeuValGlnAlaValLys
gatgacttcactggcaaatcctgcaaccagggcccttaccctcttgtccaagcagtcaag

ArgSerLeuGlySerLeu***
agaagccttggctccctgtgaattc
```

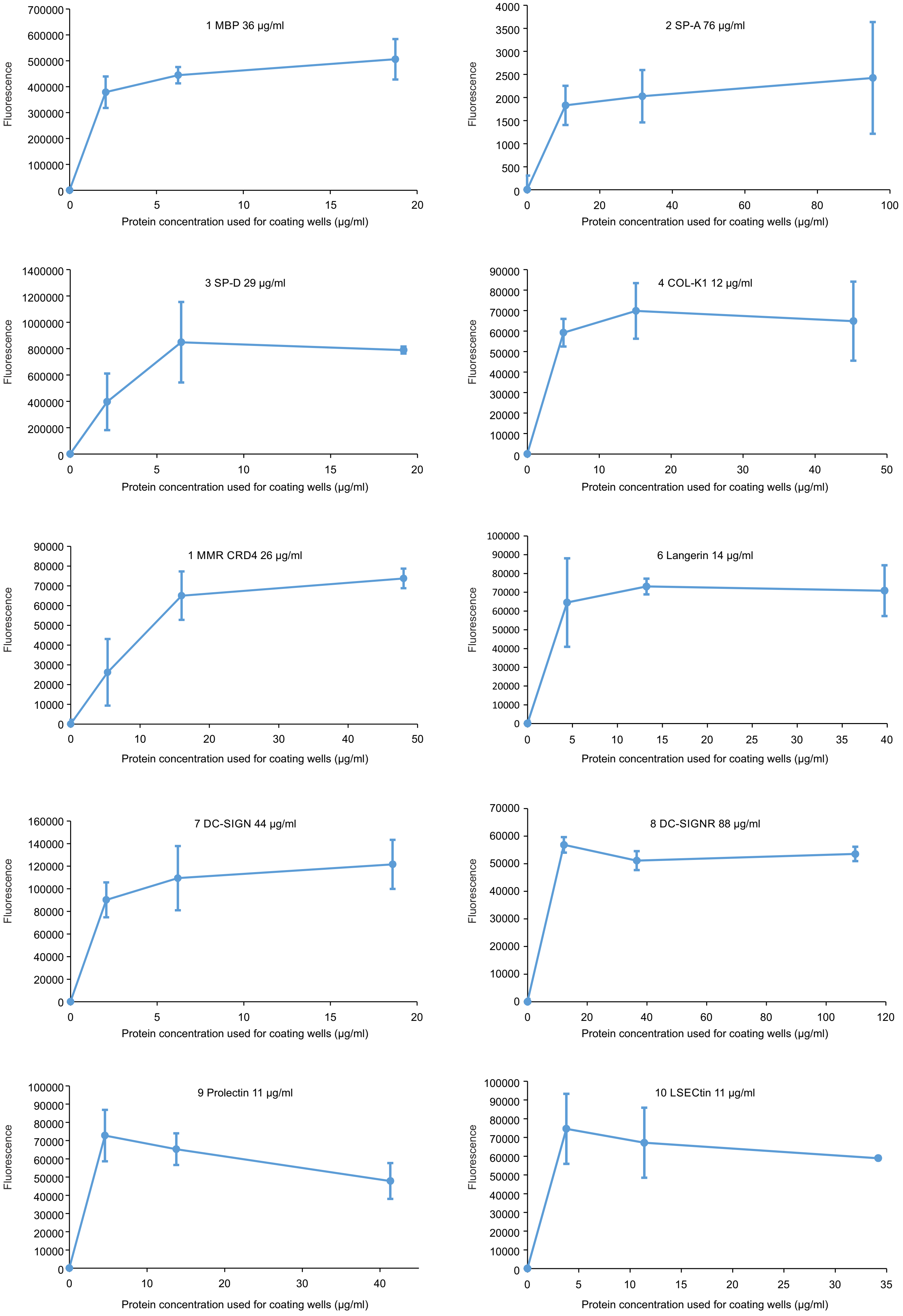

Figure S34 (Part 1). Saturation curves for coating of assay wells.
Concentrations of aliquots used to coat individual wells are indicated at the top of each panel.

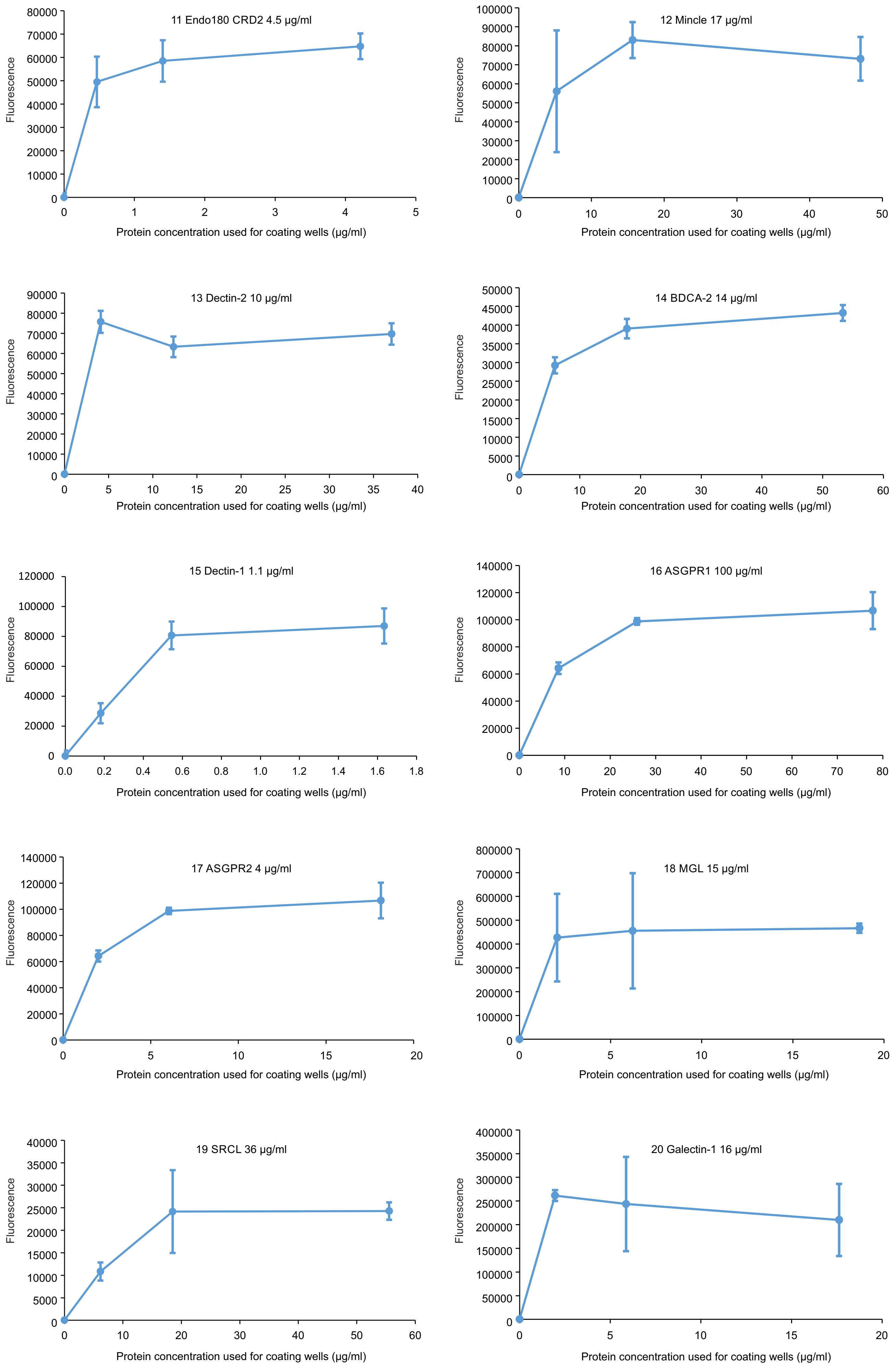

Figure S34 (Part 2). Saturation curves for coating of assay wells.
Concentrations of aliquots used to coat individual wells are indicated at the top of each panel.

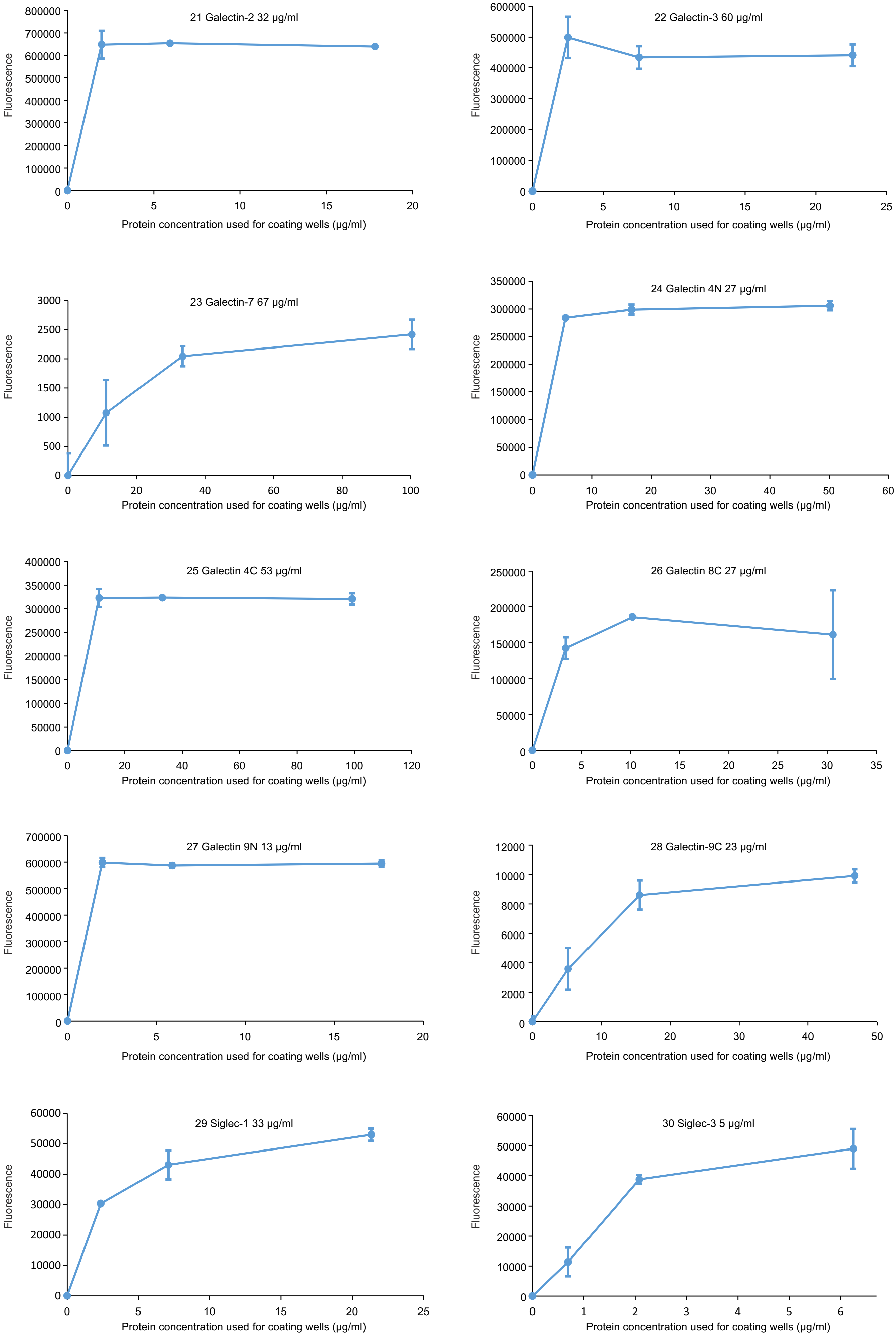

Figure S34 (Part 3). Saturation curves for coating of assay wells.
Concentrations of aliquots used to coat individual wells are indicated at the top of each panel.

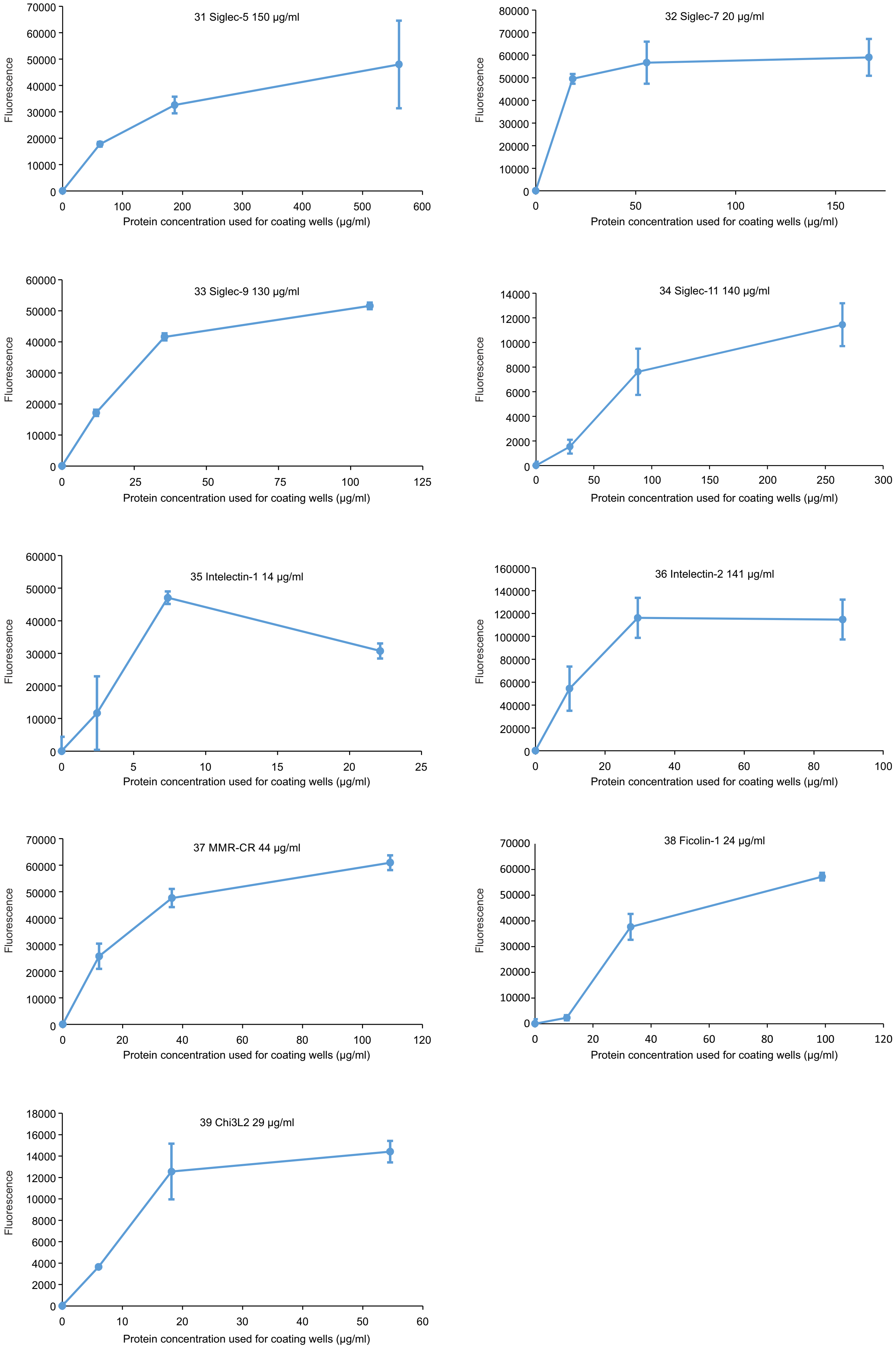

Figure S34 (Part 4). Saturation curves for coating of assay wells.
Concentrations of aliquots used to coat individual wells are indicated at the top of each panel.
